# Fecal Microbiota Transplantation Is a Promising Method to Restore Gut Microbiota Dysbiosis and Relieve Neurological Deficits after Traumatic Brain Injury

**DOI:** 10.1155/2021/5816837

**Published:** 2021-02-10

**Authors:** Donglin Du, Wei Tang, Chao Zhou, Xiaochuan Sun, Zhengqiang Wei, Jianjun Zhong, Zhijian Huang

**Affiliations:** ^1^Department of Neurosurgery, The First Affiliated Hospital of Chongqing Medical University, Chongqing 400016, China; ^2^Department of Gastrointestinal Surgery, The First Affiliated Hospital of Chongqing Medical University, Chongqing 400016, China

## Abstract

**Background:**

Traumatic brain injury (TBI) can induce persistent fluctuation in the gut microbiota makeup and abundance. The present study is aimed at determining whether fecal microbiota transplantation (FMT) can rescue microbiota changes and ameliorate neurological deficits after TBI in rats.

**Methods:**

A controlled cortical impact (CCI) model was used to simulate TBI in male Sprague-Dawley rats, and FMT was performed for 7 consecutive days. 16S ribosomal RNA (rRNA) sequencing of fecal samples was performed to analyze the effects of FMT on gut microbiota. Modified neurological severity score and Morris water maze were used to evaluate neurobehavioral functions. Metabolomics was used to screen differential metabolites from the rat serum and ipsilateral brains. The oxidative stress indices were measured in the brain.

**Results:**

TBI induced significance changes in the gut microbiome, including the alpha- and beta-bacterial diversity, as well as the microbiome composition at 8 days after TBI. On the other hand, FMT could rescue these changes and relieve neurological deficits after TBI. Metabolomics results showed that the level of trimethylamine (TMA) in feces and the level of trimethylamine N-oxide (TMAO) in the ipsilateral brain and serum was increased after TBI, while FMT decreased TMA levels in the feces, and TMAO levels in the ipsilateral brain and serum. Antioxidant enzyme methionine sulfoxide reductase A (MsrA) in the ipsilateral hippocampus was decreased after TBI but increased after FMT. In addition, FMT elevated SOD and CAT activities and GSH/GSSG ratio and diminished ROS, GSSG, and MDA levels in the ipsilateral hippocampus after TBI.

**Conclusions:**

FMT can restore gut microbiota dysbiosis and relieve neurological deficits possibly through the TMA-TMAO-MsrA signaling pathway after TBI.

## 1. Introduction

Traumatic brain injury (TBI) is a global public health concern, which affects approximately 10 million people each year worldwide [1] and is the primary cause of death in individuals under the age of 45 years [2]. More than 10^13^-10^14^ gut microorganisms from over 1000 different known bacterial species exist in the human gut, which outnumber the somatic cells in the human body, and the bacterial genome is 100-fold greater than those in the human genome [3, 4]. The gut microbiota has a close relationship with the central nervous system (CNS). An increasing number of studies have shown that the gut microbiota can regulate development of the CNS and brain function [5–7]. In addition, the gut microbiome structure and composition are altered in neurological diseases, such as Alzheimer's disease, Parkinson's disease, autism, schizophrenia, and depression. These microbiome changes can contribute to the development of disease pathology [8–12].

Treangen et al. [13] reported that TBI, an acute brain injury, can induce a decrease in the abundance of *Lactobacillus gasseri*, *Ruminococcus flavefaciens*, and *Eubacterium ventriosum* and an increase in the abundance of *Eubacterium sulci* and *Marvinbryantia formatexigens* at 24 h after TBI. Nicholson et al. [14] also showed that TBI could cause gut microbiome dysbiosis that persisted for 7 days after TBI, and this dysbiosis was correlated with lesion volume. Cerebral ischemic stroke, another acute brain injury, also induces significant gut microbiota dysbiosis, which is correlated with the injury severity and outcome [15, 16]. These findings indicate that restoring the gut microbiome could bring some therapeutic effect.

Fecal microbiota transplantation (FMT) is a method used to introduce new intestinal flora by transferring the mixture of bacteria from normal feces to the dysbiotic gastrointestinal tract. This method has been found to normalize gut microbiota dysbiosis after acute cerebral ischemic stroke and exert a neuroprotective effect [17, 18]. However, it remains unknown whether FMT can normalize the gut dysbiosis, thereby improving the neurological functional recovery after TBI. Therefore, the goals of this study were to confirm that TBI could induce significant gut microbiota dysbiosis and to determine whether FMT could rescue dysbiosis and exert therapeutic effects after TBI.

Gut microbiota can bidirectionally communicate with the brain through different routes, including metabolism, immune, and circulation routes [19]. For example, the gut microbiota can metabolize indigestible food ingredients and produce many substances, such as short-chain fatty acid (SCFA), some of which can be absorbed into the bloodstream and penetrate the blood-brain barrier [20]. The current study used the serum and brain metabolomics analyses to investigate the possible molecules that mediate communication between the gut and the brain.

## 2. Methods

### 2.1. Animals

Male Sprague-Dawley rats (8 weeks old, *n* = 177) were obtained from the laboratory animal center of Chongqing Medical University (Chongqing, China).The rats were randomly divided into four groups: the sham group (received only craniotomy, without TBI or FMT, *n* = 44), TBI group (received TBI, *n* = 17), TBI+saline group (received TBI and saline, *n* = 53), and TBI+FMT (received both TBI and FMT, *n* = 53) groups. Lastly, 10 rats without any intervention were selected as donor rats. Five rats were housed per cage, and the background diet was kept constant throughout the experiments. The diet composition was shown in Supplementary Table 1. The animals in this study were housed in a standard 12 h : 12 h light-dark cycle with food and water available ad libitum. The animal experiments in this study adhered to the Animal Research: Reporting of In Vivo Experiments and were approved by the Institutional Animal Care and Use Committee (IACUC) of Chongqing Medical University (approval no. 2019335).

### 2.2. Induction of Controlled Cortical Impact

Controlled cortical impact (CCI) was induced by a contusion device (TBI 0310; Precision Systems & Instrumentation, Fairfax Station, VA, USA) under general anesthesia via intraperitoneal injection of 3% pentobarbital sodium (50 mg/kg) according to a previous report [21]. A longitudinal midline incision about 1.5 cm was created, and a 5 mm craniotomy was produced in the right parietal skull using a portable drill. The CCI parameters were set as follows: velocity: 5.0 m/s, depth: 3.0 mm, and dwelling time: 500 ms.

### 2.3. FMT Procedures

#### 2.3.1. Preparation of Fecal Suspension

Fresh stool samples from donor rats housed under specific pathogen-free conditions were immediately collected and stored at -80°C before use. The fecal pellets were then mixed immediately in sterile 0.9% saline to 200 mg/mL, and the supernatant was collected after the suspension was centrifuged at 2000 rpm for 10 min, which was prepared once daily [17].

#### 2.3.2. Treatment with FMT

The rats were fixed in a supine position on a plate, after which sterilized catheters were inserted into the anus up to 5–8 cm. Then, 5 mL fecal suspension or equal volume of sterile 0.9% saline was injected into the colon through the catheters and maintained for 3 min [22]. Fecal microbiota transplantation or equal volume of saline transplantation was performed 2 h after TBI, for 7 consecutive days.

### 2.4. Fecal Genomic DNA Extraction and 16S Ribosomal RNA Sequencing

#### 2.4.1. Sample Collection and 16S RNA Sequencing

The fecal samples from the sham, TBI, TBI+saline, and TBI+FMT groups were collected 1 day before TBI (pre-TBI), and on 8 days after injury (*n* = 10, per group). The fecal samples from donor rats were also collected (*n* = 10). The samples were immediately collected in sterile tubes on ice and stored at -80°C. Fecal genomic DNA extraction, PCR amplification, and sequencing were conducted by Majorbio BioPharm Technology Co., Ltd. (Shanghai, China) [23, 24].The procedure of DNA extraction, PCR amplification, and sequencing are (available here) in the supplementary material.

#### 2.4.2. Processing of Sequencing Data

Raw fastq files were demultiplexed and quality filtered by Trimmomatic and merged by FLASH with the following criteria: (i) the reads were truncated at any site receiving an average quality score < 20 over a 50 bp sliding window. (ii) Primers were exactly matched allowing 2 nucleotide mismatching, and reads containing ambiguous bases were removed. (iii) Sequences whose overlap longer than 10 bp were merged according to their overlapped sequences. The UPARSE (version7.1,http://drive5.com/uparse/) and UCHIME algorithms were used to select operational taxonomic units (OTUs) at 97% sequence identity and to trim the data, respectively. The acquired OTU taxonomy was assessed against the Silva (SSU123) 16S ribosomal RNA database at a confidence threshold of 70% using the Ribosomal Database Project Classifier algorithm (http://rdp.cme.msu.edu/).

#### 2.4.3. Bioinformatics Analysis

Alpha diversity indices (*Shannon*, *Ace*, *Chao*, *Coverage*, and *Shannon even* indices) were calculated in QIIME (version 1.7.0) and examined using one-way analysis of variance (ANOVA) in SPSS 21.0. Principal coordinate analysis (PCoA) based on Bray-Curtis similarities and ANOSIM analysis was performed using the R software package (https://http://www.r-project.org/). One-way ANOVA with false discovery rate (FDR), multiple comparisons correction, and Tukey's post hoc comparisons test was calculated at the phylum, family, and genus levels among each groups and corrected *p* < 0.05 was considered statistically significant.

### 2.5. Behavioral Testing

The modified neurological severity score (mNSS) is sensitive to unilateral cortical injury, as it includes motor function, sensory function, reflex, and balance tests. Two researchers who were blinded to each group (*n* = 7 per group) used mNSS to assess the neurologic deficits on 1 day pre-injury and on days 1, 3, 7, 14, and 21 after TBI [25]. The Morris water maze (MWM) paradigm was used to detect spatial learning and memory deficits as previously described [26, 27]. Briefly, all rats were tasked to find a 13.5 cm in diameter circular black plastic platform located 1 cm below the water surface for 6 consecutive days (from days 15–20 after TBI) in a large circular tank (180 cm in diameter by 45 cm in height) filled with black cloudy water (depth of 30 cm). The spatial cues, which were placed on the walls, remained constant, and the water temperature was maintained at 25–28°C. The rats completed five trials per day from one of the four locations (i.e., north, south, east, and west) and were given 120 s to find the platform. If the rats were unable to locate the platform within 120 s, they were placed on the platform for 10 s. The rats were given a 30 s probe test after the platform was removed on the final trial on day 6. The latency and path length to find the platform were recorded with a computer (SLY-WMS Morris Water Maze System; Zhenghua Biological Equipment Co., Ltd., Huaibei, China).

### 2.6. Serum Samples

On day 8 after TBI, the whole blood of rats was collected from the inferior vena cava at the same time (09:30–11:30 am) after fasting for more than 8 h. Briefly, whole blood samples were taken with a 5 mL syringe from the inferior vena cava [28]. After laparotomy, the blood samples (~4 mL) were collected in MiniCollect® Coagulation tubes (Greiner Bio-One, Kremsmünster, Austria) with 3.8% sodium citrate and incubated for 20 min at room temperature. The serum was collected from the yellowish supernatant after centrifugation at 1300 g for 10 min, at 4°C, and immediately stored at -80°C until subsequent assay.

### 2.7. Untargeted Metabolomics Profiling

#### 2.7.1. Sample Preparation and Metabolomics Profiling

Serum and ipsilateral brains from the sham, TBI+saline, and TBI+FMT groups (*n* = 9, per group) were collected in sterile and enzyme-free tubes on ice, and the brain tissue samples were quickly frozen in liquid nitrogen. The metabolomics were analyzed at Applied Protein Technology (APT, Shanghai) [29, 30].The protocols of sample preparation, UHPLC-Q-TOF/MS metabolomics profiling, are (available here) in the supplementary material.

#### 2.7.2. Data Analysis

MetaboAnalyst 4.0 (http://www.metaboanalyst.ca) was employed for the statistical analysis [31]. Orthogonal partial least-squares discriminant analysis (OPLS-DA), a multivariate statistical analysis model, were conducted with SIMCA-P software (version 14.1, Umetrics,Umea, Sweden). OPLS-DA models were validated based on the multiple correlation coefficient (*R*^2^) and cross-validated *Q*^2^ in cross-validation and permutation tests by applying 200 iterations. A *Q*^2^ value of more than 0.3 showed that the model was stable and reliable. The permutation tests are deemed valid when all *Q*^2^ and *R*^2^ values showed gradual decline. The variable importance in the projection (VIP) value of each variable in the OPLS-DA model was calculated to indicate its contribution to the classification. Metabolites with VIP value > 1 was further applied to Student's *t*-test at univariate level to measure the significance of each metabolite; *p* values less than 0.1 were considered as statistically significant [32].

### 2.8. CAT, SOD, MDA, GSSG, and ROS Measurements

The activities of catalase (CAT), superoxide dismutase (SOD), malondialdehyde (MDA), oxidized glutathione (GSSG), and reactive oxygen species (ROS) levels were measured using commercial kits (Nanjing Jiancheng Bioengineering Institute, Nanjing, China) according to the manufacturer's Iinstructions [21, 33]. The concentration of protein was measured using the BCA kit method [34].

### 2.9. TMAO and TMA Measures

An enzyme-linked immunoassay (ELISA) quantitation kit (Jianglai, Shanghai, China) was used to detect the serum and ipsilateral brain concentration of TMAO, according to manufacturer's protocol [35]. Analysis of TMA in the feces was performed by a 7890A gas chromatography system coupled to a 5975C inert XL EI/CI mass spectrometer (Applied Protein Technology, Shanghai).

### 2.10. Serum Proteomics

#### 2.10.1. Sample Preparation and Proteomics Profiling

Serum sample from the TBI+saline and TBI+FMT groups (*n* = 9, per group) were collected in sterile tubes, and 100 *μ*L serum from each three samples was mixed together as one sample (total of 300 *μ*L). Then, the mixed sample was stored at -80°C before subsequent assay. The serum proteomics was conducted at PTM BioLabs (Hangzhou, China) [36]. The procedure of sample preparation, LC-MS/MS analysis, and protein identification and quantification are (available here) in the supplementary material.

#### 2.10.2. Data Processing and Bioinformatics Analysis

According to the relative quantitative of protein iBAQ intensity, the differential expression of proteins and the ratio of the average value between the two samples were calculated. The relative quantitative value of each sample was analyzed as log_2_, and two-sample *t*-test was used to calculate the *p* value. Differences were considered significant at a 1.5-fold cutoff and *p* value < 0.05. Wolfpsort (https://wolfpsort.hgc.jp/) was used to predict the subcellular localization of the DEPs. The enrichment analysis was derived from the Perl module (v.1.31, https://metacpan.org/pod/Text::NSP::Measures::2D::Fisher) and results compared by two-tailed Fisher's exact test.

#### 2.10.3. DEP Validation

An enzyme-linked immunoassay (ELISA) quantitation kit (Zhen Shanghai and Shanghai Industrial Co., Ltd.) was used to detect the serum concentration of selected DEPs, according to protocols recommended by the manufacturer.

### 2.11. Western Blot

Ipsilateral brains were dissected out, and hippocampal tissues were quickly removed for Western Blot, which was conducted following a standard technique as previously described [37]. Primary antibodies used were rabbit MsrA (1 : 500,Proteintech, China) and *β*-actin (1 : 5000, Affinity, China). Blots were detected by enhanced chemiluminescence and analyzed using ImageJ software (NIH, USA).

### 2.12. Statistical Analyses

The data analysis was performed using SPSS 21.0 software, and results from the behavioral tests were analyzed by two-way analysis of variance (ANOVA) followed by Tukey's post hoc test. The remaining data were evaluated by Student's *t*-test or one-way ANOVA, followed by Tukey's post hoc test [38]. A *p* < 0.05 was considered statistically significant. Figures were processed using GraphPad Prism 8 (GraphPad Software, San Diego, CA, USA) and Adobe Photoshop CS4 software (Adobe Systems Inc., San Jose, CA, USA).

## 3. Results

### 3.1. FMT Ameliorates Dysbiosis of Gut Microbiota after TBI

The result of 16sRNA sequence showed that there was no difference in the gut microbiota diversity and composition among the sham, TBI, TBI+saline, and TBI+FMT groups before surgery, which indicated that there were no biological gut microbiome differences among these groups before surgery. It also showed that the intestinal flora from donor rats was similar with the intestinal flora of rats before surgery (Supplementary Figure S1).

**Figure 1:**
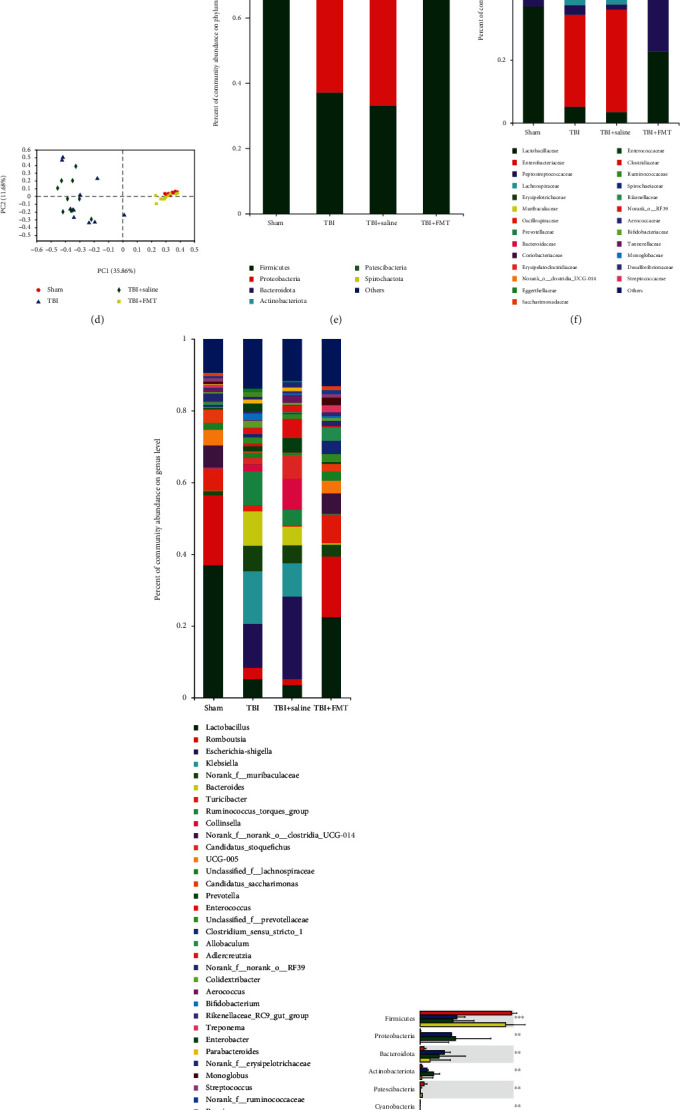
FMT can rescue the alteration of gut microbiota due to TBI. (a, b) The sequence reads and OTUs among the sham, TBI, TBI+saline, and TBI+FMT groups, at 8 days after injury. (c) Indices of *α*-diversity measured by *Shannon*, *Ace*, *Chao*, *Coverage*, and *Shannon even* in the sham, TBI, TBI+saline, and TBI+FMT groups, at 8 days after injury. (d) PCoA of the gut microbiome composition on OTU level based on the Bray-Curtis distance for FMT in TBI rats (*p* = 0.001, *R*^2^ = 0.6025). Each point indicates samples from rats of each group, which is indicated by a different color and shape. PC: principal component. (e–g) Bar plot analysis of gut microbiota relative abundance of bacterial phylum, family, and genus in the sham, TBI, TBI+saline, and TBI+FMT groups. Different colors represent different phyla, family, and genus. (h) Significant differences in the abundance of phyla in each group. (i) Significant differences in the abundance of families with high abundance (top 10). (j) Significant differences in the abundance of genus with high abundance (top 15) (*n* = 10 rats in each group). Data are presented as the mean ± SD. ^∗^*p* < 0.05, ^∗∗^*p* < 0.01, ^∗∗∗^*p* < 0.001 one-way ANOVA.

At 8 days after TBI, there were no differences in the sequence reads among each group (Figure 1(a)) and the number of OTUs on TBI and TBI+saline groups were similar but significantly less than the sham and TBI+FMT groups, indicating that the total number of taxonomic units representing microbial species had changed after 8 days post-TBI (Figure 1(b)). The *α*-diversity were measured by the *Ace*, *Chao*, *Coverage*, *Shannon*, and *Shannon even* indices. There were no differences in the value of *Ace*, *Chao*, and *Coverage indices* in the TBI group when compared with the TBI+saline group. However, the value of the indices were significant lower, when compared to the sham and TBI+FMT groups, indicating that TBI can result in decreased richness and coverage of the microbial species and that the saline did not impact community richness and coverage. On the other hand, there were no differences in the value of *Shannon* and *Shannon even* indices between the TBI and TBI+saline groups, which represent community diversity or evenness. In addition, the value of *Shannon* and *Shannon even* indices of the TBI and TBI+saline groups were relatively reduced, compared with the sham and TBI+FMT groups (Figure 1(c)). The *β*-diversity as depicted in principal coordinate components analysis (PCoA) of the Bray-Curtis and ANOSIM analysis revealed that the TBI and TBI+saline groups had a similar gut microbial community but had a different distribution of the gut microbial community when compared with the sham or TBI+FMT groups (*p* = 0.001, *R* = 0.6025; Figure 1(d)).

In the gut microbiota, there were no significant differences at the phylum, family, and genus levels between the TBI and TBI+saline groups (Figures 1(e)–1(g)), which indicated that saline did not impact composition of the gut microbial community. At the phylum level, the relative abundance of phyla *Firmicutes*, *Patescibacteria*, and *Cyanobacteria* were lower and the relative abundance of *Bacteroidetes*, *Proteobacteria*, and *Actinobacteriota* were increased in the TBI and TBI+saline groups compared to the sham or TBI+FMT groups (*p* < 0.05; Figure 1(h)). At the family level, the TBI and TBI+saline groups have 39 different bacteria when compared with the sham and TBI+FMT groups (Supplementary Table S2). Further analysis found that FMT reversed TBI-induced alterations of 8 families with high relative abundance (top 10), such as *Lactobacillaceae*, *Enterobacteriaceae*, *Peptostreptococcaceae*, *Erysipelotrichaceae*, *Oscillospiraceae*, *Muribaculaceae*, *Bacteroidaceae*, and *norank_o__Clostridia_UCG-014* (Figure 1(i)). At the genus level, the TBI group has 65 different bacteria when compared with the sham and TBI+FMT groups (Supplementary Table S3). Furthermore, stratification of genera per abundance (top 15) showed that the TBI group had a lower relative abundance of genera *Lactobacillus*, *Romboutsia*, *Turicibacter*, *norank_f__norank_o__Clostridia_UCG-014,UCG-005*, and *Candidatus_Saccharimonas* and a higher relative abundance *of genera Escherichia-Shigella*, *norank_f__Muribaculaceae*, *Bacteroides*, *Collinsella*, and *Candidatus_Stoquefichus* compared to the sham or TBI+FMT groups, which indicated that TBI can lead to multiple gut bacterial genera changes while FMT can restore the abundance of these bacterial genera (Figure 1(j)).

### 3.2. FMT Improved Neurological Behaviors

**Figure 2:**
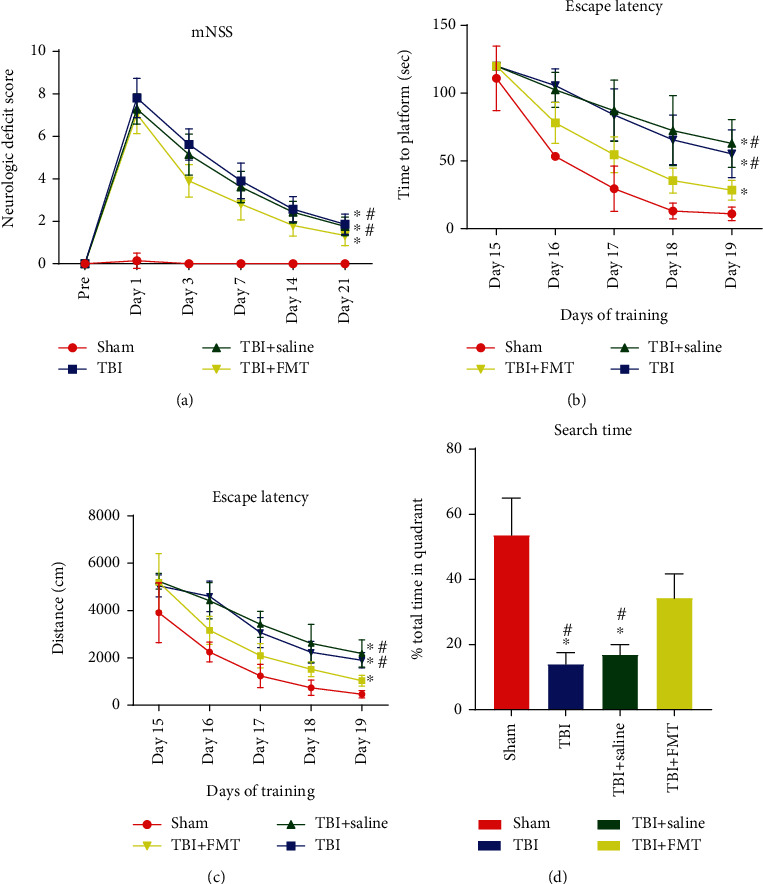
FMT ameliorates the functional outcomes after CCI. (a) Rats subjected to CCI exhibited neurologic deficits from days 1 to 21 after surgery. (b, c) Latency including time and distance spent in the MWM test to locate the submerged platform from days 15 to 19 after CCI. (d) Time spent in the correct quadrant in spatial memory performance on the final acquisition trial of training in MWM (*n* = 7 per group, mean ± SD, ^∗^*p* < 0.05 vs. the sham group, ^#^*p* < 0.05 vs. the TBI+FMT group).

The mNSS score was applied to estimate the impact of FMT on neurologic deficits. The injury groups which include TBI, TBI+saline, and TBI+FMT groups had significantly higher mNSS scores than the sham group on days 1, 3, 7, 14, and 21 after TBI. The TBI+FMT group had lower mNSS scores compared to the TBI and TBI+saline groups on days 3, 7, 14, and 21 after TBI, while there were no significant differences between the mNSS scores of the TBI and TBI+saline groups (*p* < 0.05; Figure 2(a)), which indicated that FMT brought about significant improvement of general neurological function after TBI, while saline do not improve the neurological function after TBI. The effects of FMT on learning and spatial memory were examined using the MWM test from day 15 to day 20 after TBI. The injury groups took a longer escape latency to find the hidden platform from day 16 to day 19 after TBI, compared to the sham group. The TBI and TBI+saline groups took similar time to find the hidden platform from day 16 to day 19, while the TBI+FMT group took a shorter time to find the hidden platform from day 16 to day 19 after TBI compared to the TBI and TBI+saline group (*p* < 0.05; Figures 2(b) and 2(c)), indicating that saline do not impact the learning ability after TBI and FMT could improve the learning ability after TBI. On day 20 post-TBI (the last testing day), the hidden platform was removed and the rats were expected to continue probing the platform. The injury groups spent less time in the quadrant where the platform was originally located compared to the sham group, whereas the TBI+FMT group spent more time in the quadrant compared to the TBI and TBI+saline groups, and the TBI and TBI+saline groups spent similar time in the quadrant (*p* < 0.05; Figure 2(d)), indicating that the saline did not improve the memory while FMT can ameliorate the memory after TBI.

### 3.3. Different Brain Tissues and Serum Metabolites among the Sham, TBI+Saline, and TBI+FMT Groups

**Figure 3:**
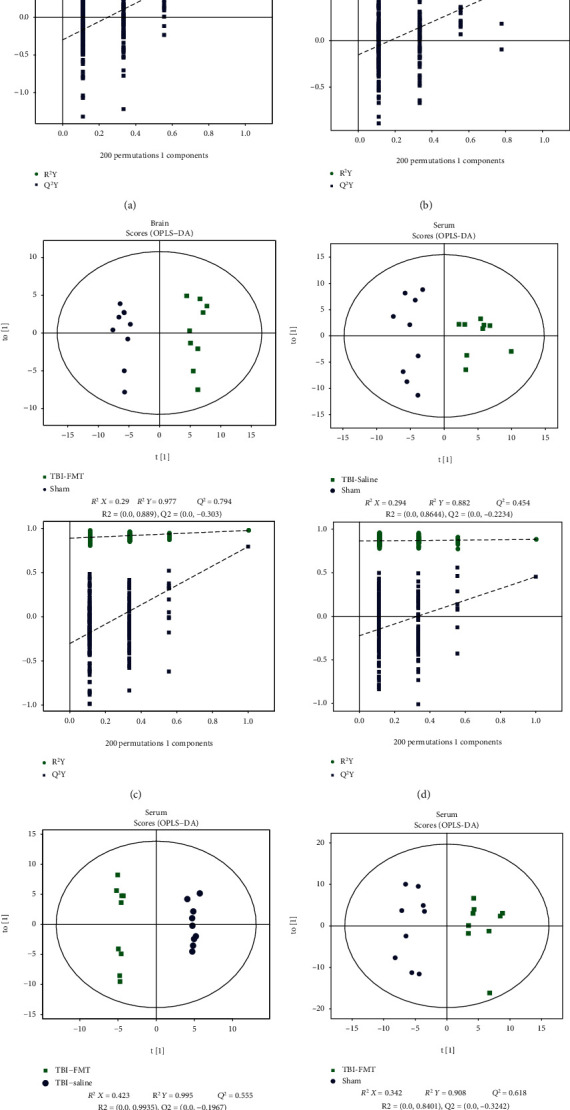
Brain and serum metabolomic analyses between the sham, TBI+saline, and TBI+FMT groups. (a–c) OPLS-DA model and permutation test of brain in the three groups. (a) TBI+saline versus sham. (b) TBI+FMT versus TBI+saline. (c) TBI+FMT versus sham. (d–f) OPLS-DA model and permutation test in serum of the three groups. (d). TBI+saline versus sham, (e) TBI+FMT versus TBI+saline. (f) TBI+FMT versus sham.

The result of 16S RNA sequence and behavioral testing showed that saline does not impact the gut microbiota and neurological behaviors after TBI; therefore, the sham, TBI+saline, and TBI+FMT groups were selected to conduct metabolomics analysis. To profile differential metabolism, the OPLS-DA analysis was performed using these three groups, which was compared in pairs and possessed satisfactory fit. The result showed a clear separation in two groups (Figures 3(a)–3(f)). The permutation tests with 200 iterations were performed, which also validated these models. In the brain, 51 metabolites were found to be differentially expressed in the sham group when compared to the TBI+saline group (Supplementary Table S4), and 32 metabolites were differentially expressed in the TBI+FMT group when compared to the TBI+saline group (Table 1). In the serum, 16 metabolites were differentially expressed in the sham when compared to the TBI+saline group (Supplementary Table S5), while 14 metabolites were differentially expressed in the TBI+FMT group when compared to the TBI+saline group (Table 2). Combing analysis of the brain and serum metabolites found that trimethylamine N-oxide (TMAO) was elevated simultaneously in brain tissues and serum of the TBI+saline group, whereas TMAO was reduced in the TBI+FMT group. The serum level of TMAO in the TBI+saline group was increased approximately 4-folds when compared to the sham group and increased approximately 3-folds when compared to the TBI+FMT group. The ipsilateral brain level of TMAO in the TBI+saline group was increased approximately 14-folds when compared to the sham group and increased approximately 6-folds when compared to the TBI+FMT group.

### 3.4. TMA and TMAO in Rats

**Figure 4:**
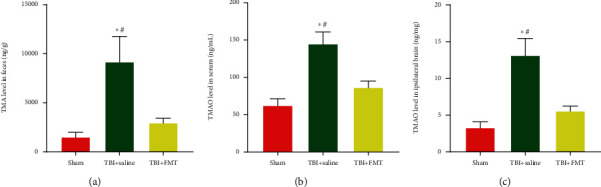
FMT decreased the level of TMAO in the serum, ipsilateral brain, and TMA in feces after TBI. (a) TMA level in feces. (b) TMAO level in the serum. (c) TMAO in the ipsilateral brain (*n* = 6 per group, mean ± SD, ^∗^*p* < 0.05 compared with the sham group, ^#^*p* < 0.05 compared with the TBI+FMT group by one-way ANOVA).

The preliminary metabolism result showed that FMT can decrease the TBI-induced increase in TMAO in serum and ipsilateral brain. TMAO, a microbiome-derived metabolite, was oxidized in the liver from a substrate known as trimethylamine (TMA), produced by the gut microbiome [39]. In order to explore the changes of TMA and TMAO after TBI, the TMA level in feces and TMAO level in serum and ipsilateral brain were measured. We found that TMA contents of the feces was significantly increased in the TBI+saline group compared to the sham group and TBI+FMT group (*p* < 0.05, Figure 4(a)). The TMAO levels in the serum and ipsilateral brain were significantly increased in the TBI+saline group, compared to the sham group and TBI+FMT group (*p* < 0.05, Figures 4(b) and 4(c)), which indicated that TBI increased the TMAO, and FMT can decrease this metabolite.

### 3.5. Serum Proteomic Analysis of the TBI+FMT and TBI+Saline Groups

**Figure 5:**
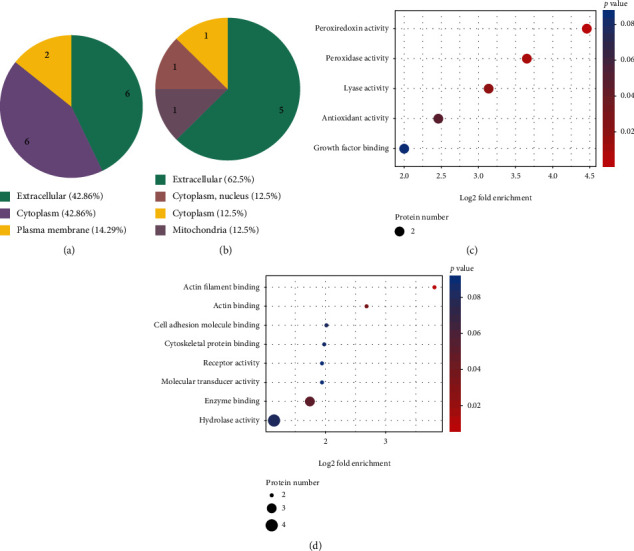
Serum proteomic analysis between the TBI+FMT and TBI+saline groups. (a) The most significantly enriched cellular components of the upregulated proteins between the TBI+FMT and TBI+saline groups. (b) The most significantly enriched cellular components of the downregulated proteins between the TBI+FMT and TBI+saline groups (c). The upregulated proteins of GO enrichment analysis of different proteins from the TBI+FMT and TBI+saline groups. (d) The downregulated proteins of GO enrichment analysis of different proteins from the TBI+FMT and TBI+saline groups.

In total, 670 proteins were identified and 527 proteins were quantified. With a 1.5-fold cutoff and *p* < 0.05 indicating significant differences, 14 proteins were found to be significantly upregulated and 8 proteins were significantly downregulated in the TBI+FMT group compared to the TBI+saline group (Supplementary Table S6). The most significantly enriched cellular components of the upregulated and downregulated proteins were extracellular components (Figures 5(a) and 5(b)). To further understand the DEPs, functional enrichment analyses were performed using Fisher's exact test *p* value(-log10(*p* value)).In the molecular functional group, the upregulated proteins were mainly involved in peroxiredoxin, peroxidase, lyase, and antioxidant activities, and the downregulated proteins were mainly involved in actin filament binding, actin binding, and enzyme binding organelles (Figures 5(c) and 5(d)).

### 3.6. Verification of the Proteomic Results by ELISA

**Figure 6:**
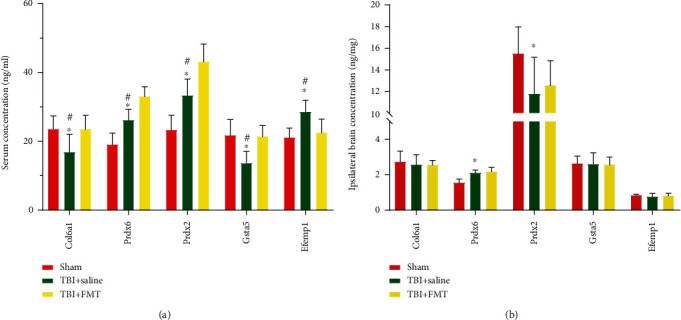
Five randomly selected DEPs concentration in the serum and ipsilateral brain. (a) The DEP concentration in serum. (b) The DEP concentration in the ipsilateral brain (*n* = 8; mean ± SD, ^∗^*p* < 0.05 compared with the sham group, ^#^*p* < 0.05 compared with the TBI+FMT group).

Five DEPs were randomly selected from the different proteins to verify the proteomics results by ELISA, and they were peroxiredoxin-6 (Prdx6), Prdx2, collagen, type VI, alpha 1 (Col6a1), glutathione S-transferase alpha 5 (Gsta5), and EGF-containing fibulin extracellular matrix protein 1 (Efemp1). As shown in Figure 5, the results revealed that the serum levels of Prdx6, Prdx2, Col6a1, and Gsta5 were significantly increased, while the serum level of Efemp1 was significantly reduced in the TBI+FMT group, compared to the TBI+saline group. In addition, the serum concentrations of Col6a1 and Gsta5 were significantly decreased and the serum concentrations of Prdx6, Prdx2, and Efemp1 were significantly increased in the TBI+saline group, compared to the sham group (Figure 6(a)).

Proteins levels in the ipsilateral brain tissues were also measured. The result showed that the levels of Prdx6 were increased, and the levels of Prdx2 was decreased in the TBI+saline group, compared with the sham group. There were no differences in the level of Col6a1, Gsta5, and Efemp1 in the TBI+saline group, compared with the sham group. These proteins in the TBI+saline group were not different from those of the TBI+FMT group. These results showed that changes of DEPs of the serum and brain tissue in TBI after FMT are not parallel (Figure 6(b)).

### 3.7. FMT Reduced the Oxidative Stress Level in the Ipsilateral Hippocampus of CCI Rats

**Figure 7:**
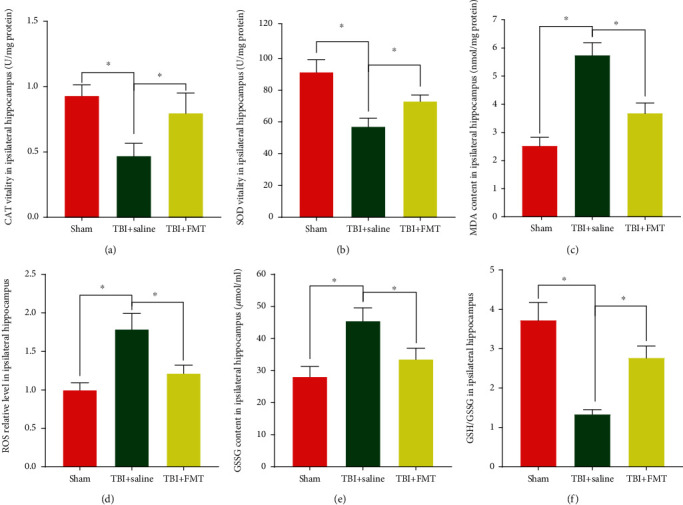
The activities of CAT and SOD and the levels of MDA and ROS, GSSG, and the GSH/GSSH ratio in the ipsilateral hippocampus. (a) CAT activity. (b) SOD activity. (c) MDA level. (d) ROS level. (e) GSSH level. (f) GSH/GSSG ratio (*n* = 4; mean ± SD, ^∗^*p* < 0.05, compared with the TBI+saline group).

The TBI+saline group showed a significant decrease in CAT and SOD activities in the ipsilateral hippocampus, compared to the sham group, while the TBI+FMT group showed significant increase in CAT and SOD activities, compared to the TBI+saline group (*p* < 0.05, Figures 7(a) and 7(b)). Higher levels of MDA were measured in the ipsilateral hippocampus of the TBI+saline group, compared to the sham group, and reduced MDA levels were found in the ipsilateral hippocampus of the TBI+FMT group, compared to the TBI+saline group (*p* < 0.05, Figure 7(c)). The TBI+saline group had significantly elevated ROS and GSSG levels and reduced GSH/GSSG ratio in the ipsilateral hippocampus, compared to the sham group, while the TBI+FMT group showed significantly reduced ROS and GSSG levels and increased GSH/GSSG ratio compared to the TBI+saline group (*p* < 0.05, Figures 7(d)–7(f)), all of which indicated that FMT can reduce oxidative stress after TBI.

### 3.8. FMT Increased MsrA Expression in the Hippocampus

**Figure 8:**
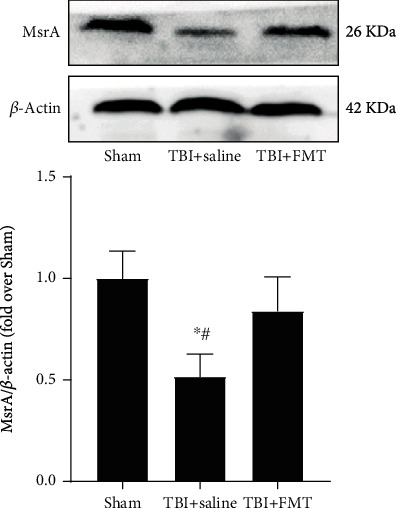
FMT increased the expression of MsrA in the hippocampus after TBI. Data are mean ± SD (*n* = 4 for each group). ^∗^*p* < 0.05, compared with sham; ^#^*p* < 0.05, compared with the TBI+FMT group.

The expression of MsrA, an intracellular enzyme that reverses protein methionine oxidation, was decreased by TMAO in the hippocampus [40]. In order to detect the changes of MsrA in TBI after FMT, Western Blot was conducted. The result showed that the MsrA expression of the TBI+saline group in the ipsilateral hippocampus was decreased, compared with the sham and TBI+FMT group, which indicated that FMT can increase the MsrA expression in the ipsilateral hippocampus (Figures 8(a) and 8(b)).

## 4. Discussion

A previous study found that TBI can cause gut microbiome dysbiosis that persisted for 7 days [14]. This study reported that TBI could cause various microbiota genus alterations at 8 days after TBI, such as genera *Lactobacillus*, *Romboutsia*, and *Turicibacter*. *Lactobacillus*, *Romboutsia*, and *Turicibacter* are SCFA-producing bacteria that were found to be decreased after TBI in this study [41–43]. The SCFA, namely, butyrate, acetate, and propionate, was reported to modulate neuroinflammation by regulating maturation and function of microglia [44]. The increased *Bacteroides*, *Escherichia-Shigella*, and *Collinsella* were also found to be associated with elevating immunoinflammatory processes [45–47]. These studies showed that the TBI-induced gut microbiome alteration may involve the immunoinflammatory process, which is a major cause of secondary TBI [48].

FMT is the most effective option to modulate gut microbiota, which has been verified for treating patients with *Clostridium difficile* infection [49, 50]. It has also been reported that FMT can normalize altered gut microbiota and ameliorate the neurological epilepsy symptom of Crohn's disease and the symptoms of autism [51, 52]. Experimental studies showed that FMT can improve the neurological function in the model of multiple sclerosis, Parkinson's disease, and stroke [9, 17, 53, 54]. In this study, we showed that FMT can rescue the gut microbial flora change and exert therapeutic effects after TBI.

Previous studies have shown that the intestinal microbiota dysbiosis after stroke can contribute to neuroinflammation via the immune pathway, and FMT can normalize the intestinal microbiota and suppress neuroinflammation by reducing proinflammatory T cells in the gut and brain [17]. Studies have also shown that FMT can reduce the activation of microglia or astrocytes in a Parkinson's disease model [9, 54], which indicated that FMT ameliorates neurological function of neurological disease via the immune pathway. In fact, the gut microorganisms communicate with the brain through the gut microbiome-derived metabolites. Intestinal flora can transform dietary components and produce some substances, which can enter the central nervous system via the blood circulation to exert biological effects [55]. The brain and serum metabolomics results showed that the level of TMAO, a gut microbiome-derived metabolite, was increased in the ipsilateral brain and serum, after TBI, and decreased after FMT.

Trimethylamine N-oxide was synthesized via a two-step process. First, gut microbes enzymatically generate TMA from choline or L-carnitine rich diet [56]. TMA, prominently produced by the intestinal microbiome [57], then enters the circulation and is further oxidized to TMAO by the hepatic enzyme, flavin-containing monooxygenase 1 and 3 (FMO1 and FMO3, primarily FMO3) [58]. Several families of bacteria such as *Deferribacteraceae*, *Anaeroplasmataceae*, *Prevotellaceae*, and *Enterobacteriaceae* are involved in TMA/TMAO production [39, 59], the latter of which was increased after TBI and decreased after FMT in the present study. The speculation that FMT decrease the level of TMAO by reducing the relative abundance of *Enterobacteriaceae* after TBI is critical, and further research is needed.

Trimethylamine N-oxide is associated with not only cardiovascular and metabolic diseases [39, 60] but also with neurological diseases, such as Alzheimer's disease, brain aging, and ischemic brain injury [61–63]. A study found that elevated circulating TMAO promotes inflammatory response and ROS production in the peripheral tissues including the aorta, heart, and kidney, contributing to the development of multiple cardiovascular and renal diseases [64–66]. Serum TMAO can cross the blood-brain barrier and lead to oxidative stress, causing damage in the brain [67, 68]. Experimental studies showed that increased circulating TMAO can increase oxidative stress by downregulating the antioxidant enzyme methionine sulfoxide reductase A (MsrA) in the hippocampus [40, 69]. MsrA, an intracellular enzyme that reverses protein methionine oxidation, has been suggested to participate in learning and memory [70]. In this study, it was shown that the oxidative stress level was increased and the expression of MsrA was decreased in the ipsilateral hippocampus after TBI, while FMT can decrease the oxidative stress level and increase the expression of MsrA. This indicated that FMT exert neuroprotective effects possibly through decreasing the level of TMAO and upregulating the antioxidant enzyme MsrA in the hippocampus.

Analysis of the serum proteomics data found that the DEPs could be involved in several molecular functions, including antioxidant activities. In this study, FMT could increase the serum levels of Prdx6, Prdx2, and Gsta5, but not the levels in the ipsilateral brain. Prdx6 and Prdx2 are members of the Prdx subfamily, which are antioxidant enzymes that hydrolyze the hydrogen peroxide generated during injury or disease. Gsta5 is a member of the glutathione-S-transferase family, which is responsible for catalyzing the conjugation of glutathione to various electrophilic compounds [71]. It has been found that Gsta5 can protect against hydrogen peroxide injury [72]. Studies showed that Prdx6 and Prdx2 exert a protective role in the liver, kidney, and heart injury via decreasing oxidative stress damage [73–76]. TBI can lead to other organ dysfunction, such as cardiac, renal, and liver diseases [77]. FMT increased the antioxidant protein level in serum, not in the ipsilateral brain, which indicated that FMT may ameliorate multiple organ damage by decreasing the oxidative stress after TBI; however, further study is needed.

TBI is a severe injury and there is no effective therapeutic method that can be used in current clinical scenario [78]. Our study showed that FMT might be a promising therapy for TBI. For FMT, there are some different approaches for administering fecal suspension, such as via enema or gavage. Considering that the majority of gut bacteria of the human body is mainly harbored in the colon [79], we administered a fecal suspension through enema, which could directly achieve a colonization effect; however, it will take a longer time. In some other studies, fecal suspension was administered through gavage [17, 54], which is relatively simple and take less time in contrast to procedures via enema. However, some bacteria might be killed or at least the bacterial activity might be inhibited by digestive fluids when they pass through the digestive tract.

In conclusion, the current study showed that TBI can induce the gut microbiome dysbiosis and that FMT can rescue gut microbiota dysbiosis, relieve the neurological deficits, and exert an antioxidation effect through decreasing TBI-induced TMAO and increasing the antioxidant enzyme MsrA expression in the hippocampus.

## Figures and Tables

**Table 1:** The significantly differential brain metabolites among the sham, TBI+FMT, and TBI+saline groups.

Metabolites	Rt (s)	*m*/*z*	VIP (a)	FC (a)	*p* value (a)	VIP (b)	FC (b)	*p* value (b)	VIP (c)	FC (c)	*p* value (c)
Creatinine	173.850	114.065	3.615	1.256	<0.001	3.112	0.871	0.002	2.466	1.094	0.015
Larixinic acid	478.552	127.037	3.255	3.331	<0.001	2.849	0.620	0.002	2.839	2.065	<0.001
L-Carnitine	361.106	162.112	44.016	2.859	<0.001	31.973	0.741	0.002	44.602	2.117	<0.001
Vanillin	469.473	219.004	1.757	3.378	<0.001	1.638	0.561	0.003	1.317	1.896	0.001
Trimethylamine N-oxide	333.965	76.075	1.928	14.146	0.001	2.372	0.162	0.003	0.753	2.290	0.003
D-Ribulose 5-phosphate	449.107	213.014	1.943	2.618	<0.001	1.648	0.673	0.003	1.717	1.761	<0.001
1-Palmitoyl-sn-glycero-3-phosphocholine	193.076	496.340	3.567	1.074	0.021	8.561	0.911	0.004	4.497	0.978	0.517
Deoxycytidine	210.869	228.097	4.181	2.139	<0.001	3.881	0.636	0.005	3.109	1.361	0.018
Cytidine	243.551	244.093	1.250	1.176	0.148	2.973	0.754	0.009	2.184	0.886	0.259
2-Methylbutyroylcarnitine	241.795	246.169	12.788	39.366	<0.001	12.428	0.321	0.009	8.338	12.638	0.014
D-Glucose 6-phosphate	478.474	278.062	2.040	2.508	<0.001	1.824	0.651	0.011	1.570	1.632	0.003
Thioetheramide-PC	90.949	758.569	9.220	1.702	0.001	9.813	0.701	0.015	5.630	1.193	0.016
Tyramine	277.753	120.079	4.366	1.741	0.002	4.780	0.651	0.016	2.757	1.133	0.482
Alpha-linolenic acid	35.787	296.257	0.164	1.032	0.821	3.396	0.784	0.016	3.151	0.809	0.127
Alpha-D-glucose 1-phosphate	478.397	243.025	5.497	3.558	<0.001	4.897	0.574	0.020	4.622	2.044	<0.001
Xanthine	225.039	153.039	1.270	1.392	<0.001	1.020	0.837	0.020	0.978	1.166	0.011
Stearoylcarnitine	149.761	428.371	3.584	1.985	<0.001	2.941	0.690	0.021	2.553	1.369	0.021
4-Aminobutyric acid	376.883	104.070	4.029	0.830	<0.001	3.377	1.092	0.022	3.151	0.907	0.005
1-Palmitoyl-2-hydroxy-sn-glycero-3-phosphoethanolamine	200.133	454.292	0.214	1.046	0.514	1.626	0.861	0.023	1.697	0.900	0.109
L-Pyroglutamic acid	424.879	147.076	3.476	1.266	0.002	2.784	0.879	0.028	2.621	1.112	0.029
PC(16 : 0/16 : 0)	147.838	734.569	7.800	0.752	0.022	10.741	1.282	0.035	8.232	0.963	0.749
Acetylcarnitine	333.570	204.122	2.209	6.347	<0.001	1.607	0.579	0.037	1.930	3.676	0.009
4-Hydroxybutanoic acid lactone	376.945	87.044	6.441	0.759	<0.001	4.698	1.104	0.045	6.090	0.838	0.001
O-Acetyl-L-serine	94.826	148.059	1.205	0.696	0.005	1.141	1.307	0.046	0.455	0.909	0.296
Gamma-L-glutamyl-L-glutamic acid	467.985	277.103	1.781	0.811	0.120	2.644	1.225	0.048	1.675	0.993	0.958
L-Palmitoylcarnitine	172.744	400.341	8.693	2.961	<0.001	6.577	0.662	0.049	7.956	1.960	0.005
1-Stearoyl-2-oleoyl-sn-glycerol 3-phosphocholine (SOPC)	59.082	832.584	3.308	0.952	0.619	7.163	1.226	0.064	6.379	1.167	0.038
N-Oleoylethanolamine	36.069	326.304	3.157	0.691	0.003	2.685	1.259	0.064	2.487	0.870	0.247
D-Mannose-6-phosphate	477.920	261.036	5.310	2.733	<0.001	3.547	0.720	0.074	5.190	1.968	0.001
(3-Carboxypropyl)trimethylammonium cation	380.851	146.117	8.428	1.412	0.001	5.305	0.856	0.087	7.175	1.209	0.026
Cyclohexylamine	388.461	160.132	9.374	6.809	0.004	8.565	0.497	0.087	8.296	3.383	0.005
Pantothenate	280.393	220.116	1.021	1.171	0.036	1.030	0.870	0.097	0.544	1.018	0.789

Abbreviations: FC: fold change; *m*/*z*: mass-to-charge ratio; Rt: retention time; VIP: variable importance in the projection. VIP (a) or FC (a): TBI+saline versus the sham group; VIP (b) or FC (b): TBI+FMT versus the TBI+saline group; VIP (c) or FC (c): TBI+FMT versus the sham group.

**Table 2:** The significantly differential serum metabolites among the sham, TBI+FMT, and TBI+saline groups.

Metabolites	Rt (s)	*m*/*z*	VIP (a)	FC (a)	*p* value (a)	VIP (b)	FC (b)	*p* value (b)	VIP (c)	FC (c)	*p* value (c)
1-O-(cis-9-Octadecenyl)-2-O-acetyl-sn-glycero-3-phosphocholine	189.098	550.384	2.461	0.822	0.037	2.890	1.326	0.001	0.891	1.091	0.256
L-Palmitoylcarnitine	174.714	400.341	1.956	0.851	0.106	2.651	1.246	0.014	0.161	1.060	0.500
L-Citrulline	444.719	176.101	1.082	0.680	0.005	1.089	1.435	0.014	0.142	0.976	0.826
1-Palmitoyllysophosphatidylcholine	189.717	538.384	1.891	0.816	0.035	1.800	1.163	0.022	0.741	0.949	0.560
Sphingomyelin (d18 : 1/18 : 0)	139.739	794.603	1.618	1.087	0.340	2.504	1.144	0.024	3.274	1.244	0.005
Sarcosine	317.225	131.080	0.525	1.031	0.850	1.200	0.726	0.024	0.740	0.748	0.108
Glycochenodeoxycholate	215.888	450.318	1.410	3.327	0.003	1.307	0.497	0.037	0.327	1.652	0.126
L-Phenylalanine	282.802	166.084	1.181	1.174	0.022	2.256	0.790	0.040	0.716	0.849	0.267
L-Alanine	373.579	90.054	2.132	0.326	0.056	1.561	0.715	0.043	1.237	0.759	0.099
Dioctyl phthalate	233.342	391.282	0.761	0.680	0.086	1.141	1.879	0.046	0.434	1.277	0.401
1-Stearoyl-sn-glycerol 3-phosphocholine	213.909	524.368	1.262	0.958	0.738	1.506	0.847	0.054	1.293	0.811	0.136
Trimethylamine N-oxide	333.593	76.074	1.953	3.936	0.035	1.477	0.341	0.062	0.436	1.343	0.442
Cortexolone	48.584	347.219	0.416	0.874	0.483	1.064	0.655	0.075	0.934	0.572	0.013
5-Methylcytosine	207.507	126.065	0.251	0.928	0.595	1.090	1.236	0.087	0.930	1.146	0.186

Abbreviations: FC: fold change; *m*/*z*: mass-to-charge ratio; Rt: retention time; VIP: variable importance in the projection. VIP (a) or FC (a): TBI+saline versus the sham group; VIP (b) or FC (b): TBI+FMT versus the TBI+saline group; VIP (c) or FC (c): TBI+FMT versus the sham group.

## Data Availability

The 16SRNA raw reads generated were deposited into the NCBI Sequence ReadArchive (SRA) database (Accession Number: SRP 296590).Other raw data, including metabolomics data, used to support the findings of this study are available from the first author and corresponding author upon request.

## Supplementary Materials

Supplementary 1 Materials and methods, 16S ribosomal RNA sequencing, DNA extraction, PCR amplification, and sequencing. The microbial genomic DNA of frozen fecal samples was extracted using the E.Z.N.A.® Soil DNA Kit (Omega Bio-Tek, Norcross, GA, USA), and DNA concentration and purification were measured using the NanoDrop 2000 spectrophotometer (Thermo Fisher Scientific, Wilmington, DE, USA). Electrophoresis on a 1% agarose gel was used to verify the DNA quality. The V3-V4 hypervariable regions were PCR-amplified using primers 338F (5′-ACTCCTACGGGAGGCAGCAG-3′) and 806R (5′-GGACTACHVGGGTWTCTAAT-3′). The PCR reactions were conducted using the following program: 3 min for denaturation at 95°C, 27 cycles of 30 s at 95°C, 30 s for annealing at 55°C, 45 s for elongation at 72°C, and a final extension at 72°C for 10 min. PCR reactions were performed in triplicate 20 *μ*L mixture containing 4 *μ*L of 5× FastPfu Buffer, 2 *μ*L of 2.5 mM dNTPs, 0.8 *μ*L of each primer (5 *μ*M), 0.4 *μ*L of FastPfu Polymerase, and 10 ng of template DNA and deionized distilled water for a total volume of 20 *μ*L. The amplicon products were loaded on a 2% prestained agarose gel (Thermo Fisher Scientific), and the expected fragments were extracted using QuantiFluor™-ST (Promega, Fitchburg, WI, USA). The final concentration was determined using the Qubit 1X dsDNA HS Assay Kit (Life Technologies, Bleiswijk, Netherlands), and the purified amplicons were normalized and pooled for 2 × 300 cycles of Illumina MiSeq deep sequencing (Illumina, San Diego, CA, USA). Metabolomics profiling. Sample preparation. Serum and ipsilateral brains from the sham, TBI+saline, and TBI+FMT groups (*n* = 9, per group) were collected in sterile and enzyme-free tubes on ice and the brain tissue samples were quickly frozen in liquid nitrogen. The serum sample were thawed in an ice bath, and 150 *μ*L serum sample was pipetted into a precooled microhigh-speed centrifuge tube and mixed with 400 *μ*L prechilled methanol/acetonitrile (*v*/*v* = 1/1) and centrifuged at 14,000 g, 4°C for 20 min. The supernatant was dried in a vacuum centrifuge and redissolved in 100 *μ*L acetonitrile/water (1 : 1, *v*/*v*) solvent for the UHPLC-Q-TOF/MS analysis. The brain tissues were cut on dry ice (~10 mg) and transferred into an Eppendorf tube with 200 *μ*L of H_2_O and five ceramic beads for homogenization. Metabolite extraction was performed by adding 800 *μ*L of methanol/acetonitrile (1 : 1, *v*/*v*) to the homogenized solution and centrifuged for 15 min (14000 g, 4°C). The supernatant was dried and redissolved in 100 *μ*L acetonitrile/water (1 : 1, *v*/*v*) solvent for UHPLC-Q-TOF/MS analysis. UHPLC-Q-TOF/MS analysis. The prepared sample solution were analyzed using a 2.1 mm × 100 mm ACQUIY UPLC BEH 1.7 *μ*m column. Column temperature was set at 25°C for operation, and flow rate was 500 *μ*L/min. The mobile phase consisted of mobile phase A (H_2_O+ 25 mM ammonium acetate+25 mM ammonium hydroxide) and mobile phase B (acetonitrile). The gradient was as follows: 95% B from 0 to 0.5 min, 95%–65% B from 0.5 to 7 min, 65%–40% B from 7 to 8 min, B was held at 40% from 8 to 9 min, 40%-95% B from 9 to 9.1 min, and B was held at 95% from 9.1 to 12 min for equilibration. The sample temperature was maintained at 4°C throughout the analyses. Electrospray ionization source conditions on triple TOF were set as follows: ion source Gas1 (Gas1) as 60, ion source Gas2 (Gas2) as 60, curtain gas (CUR) as 30, source temperature: 600°C, ion spray voltage floating (ISVF) ± 5500 V. In MS only acquisition, the instrument was set to acquire over the *m*/*z* range 60-1000 Da, and the accumulation time for TOF MS scan was set at 0.20 s/spectra. In auto MS/MS acquisition, the instrument was set to acquire over the *m*/*z* range 25-1000 Da, and the accumulation time for product ion scan was set at 0.05 s/spectra. Information-dependent acquisition (IDA), an artificial intelligence-based product ion scan mode, was used to detect and identify MS/MS spectra. The parameters were set as follows: declustering potential, 60 V (+) and-60 V (-); collision energy, 50 V(±15); exclude isotopes within 4 Da, candidate ions to monitor per cycle: 10. The raw MS data generated by UPLC-Q-TOF/MS were converted to MzXML files using ProteoWizard MSConvert and then imported into XCMS software for peak alignment, retention time adjustment, and extraction of peak intensities. Compound identification of metabolites was performed by comparing the accuracy of *m*/*z* value (<25 ppm), and MS/MS spectra with an in-house database established with available authentic standards. Proteomics. Sample preparation for MS. Serum samples from the TBI+saline and TBI+FMT groups were taken from -80°C, rewarmed, and centrifuged at 12000 g for 10 min, at 4°C. The supernatant was transferred to a new centrifuge tube, and the highly abundant proteins were depleted using the Seppro Rat Spin Columns Kit (Thermo Fisher Scientific). The protein concentrations were determined by the bicinchoninic acid assay, and the peptides were fractionated by high pH reverse-phase high-performance liquid chromatography (HPLC) using Agilent 300 Extend C18 column (5 *μ*m particles, 4.6 mm ID, and 250 mm length; Agilent Technologies) after trypsin digestion. The operation parameters were as follows: the peptide gradient was 8–32% acetonitrile, pH was 9, the 60 components were separated for a duration of 60 min, and the peptides were combined into four components and dried by vacuum centrifugation. 2.9.2 LC-MS/MS analysis The trypsin-digested peptides were dissolved in 0.1% formic acid (solvent A) and separated using the EASY-nLC 1000 ultra-HPLC system. Solvent B included an increase from 4% to 8% solvent (0.1% formic acid in 90% acetonitrile) for 5 min, 8–22% for 5–45 min, 22–30% for 45–55 min, 30–90% for 55–57 min, and 90% for 60 min, at a constant flow rate of 400 nL/min. The separated peptides were injected into a nanoelectrospray ionization source for ionization and analysis by Orbitrap Fusion Lumos Mass Spectrometer. The electrospray voltage was 2.0 kV, and peptide precursor ions and their secondary fragments were detected and analyzed by high-resolution Orbitrap MS. The primary MS scan range was set to 385–1500 *m*/*z*, the scan resolution was set to 60,000, and the secondary MS scan range was set to a fixed starting point of 100 *m*/*z* with a resolution set to 15,000. The data acquisition mode used a data-dependent procedure, namely, the first 20 peptide precursor ions with the highest signal strength entered into the higher energy collision dissociation collision cell and fragmented with 30% fragmentation energy, followed by sequential MS analysis. The parameters used for MS were as follows: automatic gain control of 5E4, signal threshold of 15000 ions/s, maximum injection of 30 ms, and dynamic exclusion time for tandem mass scans of 30 s to avoid repeated scans of precursor ions. Protein identification and relative quantification. Raw MS files were analyzed using the Maxquant search engine (v.1.5.2.8) and searched against the Rattus norvegicus UniProt database concatenated with the reverse decoy database. Label-free quantitation was conducted using the intensity-based absolute quantification (iBAQ) method. A maximum of two trypsin/P missing cleavages was allowed. The minimum length of peptide and maximum number of peptide modifications were set to 5 and 7, respectively. In the first search and main search, the mass tolerance for precursor ions was set at 20 and 5 ppm, respectively, and the mass tolerance for fragment ions was set to 0.02 Da. The alkylation of cysteine was specified as fixed modification and oxidation of methionine, and acetylation modification and deamination of the N-terminus of proteins were specified as variable modifications. The false discovery rate was set to <1%. Result. Figure S1: the gut microbiota of each group before surgery. A, B The sequence reads and OTUs of each group before injury. C, D *α*-Diversity and *β*-diversity of the rats before injury. E–G Bar plot analysis of gut microbiota relative abundance of bacterial phyla, family, and genus in the rats before surgery. Different colors represent different phyla, family, and genus.

Supplementary 2 Table S1 The diet composition. Table S2: significant changes of the microbiome at family level among sham, TBI, TBI+saline, and TBI+FMT groups. Table S3: significant changes of the microbiome at genus level among sham, TBI, TBI+saline, and TBI+FMT groups. Table S4: the significantly differential brain metabolites between TBI+saline and sham groups. Table S5: the significantly differential serum metabolites between the TBI+saline and sham groups. Abbreviations: FC: fold change; *m*/*z*: mass-to-charge ratio; Rt: retention time; VIP: variable importance in the projection. Table S6: the significantly differential serum protein between the TBI+saline and TBI+FMT groups.
