# Supplementary material for: Fecal Microbiota Transplantation Is a Promising Method to Restore Gut Microbiota Dysbiosis and Relieve Neurological Deficits after Traumatic Brain Injury
Source: Oxid Med Cell Longev. 2021 Feb 10;2021:5816837. doi: 10.1155/2021/5816837 (PMC7894052; doi:10.1155/2021/5816837)
Supplement: Supplementary 1 — Materials and methods, 16S ribosomal RNA sequencing, DNA extraction, PCR amplification, and sequencing. The microbial genomic DNA of frozen fecal samples was extracted using the E.Z.N.A.® Soil DNA Kit (Omega Bio-Tek, Norcross, GA, USA), and DNA concentration and purification were measured using the NanoDrop 2000 spectrophotometer (Thermo Fisher Scientific, Wilmington, DE, USA). Electrophoresis on a 1% agarose gel was used to verify the DNA quality. The V3-V4 hypervariable regions were PCR-amplified using primers 338F (5′-ACTCCTACGGGAGGCAGCAG-3′) and 806R (5′-GGACTACHVGGGTWTCTAAT-3′). The PCR reactions were conducted using the following program: 3 min for denaturation at 95°C, 27 cycles of 30 s at 95°C, 30 s for annealing at 55°C, 45 s for elongation at 72°C, and a final extension at 72°C for 10 min. PCR reactions were performed in triplicate 20 μL mixture containing 4 μL of 5× FastPfu Buffer, 2 μL of 2.5 mM dNTPs, 0.8 μL of each primer (5 μM), 0.4 μL of FastPfu Polymerase, and 10 ng of template DNA and deionized distilled water for a total volume of 20 μL. The amplicon products were loaded on a 2% prestained agarose gel (Thermo Fisher Scientific), and the expected fragments were extracted using QuantiFluor™-ST (Promega, Fitchburg, WI, USA). The final concentration was determined using the Qubit 1X dsDNA HS Assay Kit (Life Technologies, Bleiswijk, Netherlands), and the purified amplicons were normalized and pooled for 2 × 300 cycles of Illumina MiSeq deep sequencing (Illumina, San Diego, CA, USA). Metabolomics profiling. Sample preparation. Serum and ipsilateral brains from the sham, TBI+saline, and TBI+FMT groups (n = 9, per group) were collected in sterile and enzyme-free tubes on ice and the brain tissue samples were quickly frozen in liquid nitrogen. The serum sample were thawed in an ice bath, and 150 μL serum sample was pipetted into a precooled microhigh-speed centrifuge tube and mixed with 400 μL prechilled methanol/acetonitrile (v/v = 1/1) and centrifuged at [file 5816837.f1.docx]

Supplementary description:

Fig. S1. The gut microbiota of each group before surgery. A-B. The sequence reads and OTUs of each group before injury. C-D. α-diversity and β-diversity of the rats before injury. E-G. Bar plot analysis of gut microbiota relative abundance of bacterial phyla, family and genus in the rats before surgery. Different colors represent different phyla, family and genus.

Table S1. The diet composition.

Table S2. Significant changes of the microbiome at family level among Sham, TBI, TBI+saline and TBI+FMT groups.

Table S3. Significant changes of the microbiome at genus level among Sham, TBI, TBI+saline and TBI+FMT groups.

Table S4. The significantly differential brain metabolites between TBI+saline and Sham groups.

Table S5. The significantly differential serum metabolites between TBI+saline and Sham groups.

Table S6. The significantly differential serum protein between TBI+saline and TBI+FMT groups.
