# Supplementary material for: Fecal Microbiota Transplantation Is a Promising Method to Restore Gut Microbiota Dysbiosis and Relieve Neurological Deficits after Traumatic Brain Injury
Source: Oxid Med Cell Longev. 2021 Feb 10;2021:5816837. doi: 10.1155/2021/5816837 (PMC7894052; doi:10.1155/2021/5816837)
Supplement: Supplementary 2 — Table S1 The diet composition. Table S2: significant changes of the microbiome at family level among sham, TBI, TBI+saline, and TBI+FMT groups. Table S3: significant changes of the microbiome at genus level among sham, TBI, TBI+saline, and TBI+FMT groups. Table S4: the significantly differential brain metabolites between TBI+saline and sham groups. Table S5: the significantly differential serum metabolites between the TBI+saline and sham groups. Abbreviations: FC: fold change; m/z: mass-to-charge ratio; Rt: retention time; VIP: variable importance in the projection. Table S6: the significantly differential serum protein between the TBI+saline and TBI+FMT groups. [file 5816837.f2.docx]

**Supplementary Material**

**Materials and methods**

**16S ribosomal RNA sequencing**

**DNA extraction, PCR amplification, and sequencing**

The microbial genomic DNA of frozen fecal samples was extracted using the E.Z.N.A.® Soil DNA Kit (Omega Bio-Tek, Norcross, GA, USA), and DNA concentration and purification were measured using the NanoDrop 2000 spectrophotometer (Thermo Fisher Scientific, Wilmington, DE, USA). Electrophoresis on a 1% agarose gel was used to verify the DNA quality. The V3-V4 hypervariable regions were PCR-amplified using primers 338F (5’-ACTCCTACGGGAGGCAGCAG-3’) and 806R (5’-GGACTACHVGGGTWTCTAAT-3’). The PCR reactions were conducted using the following program: 3 min for denaturation at 95 °C,27 cycles of 30 s at 95 °C, 30s for annealing at 55 °C, and 45s for elongation at 72 °C, and a final extension at 72 °C for 10 min. PCR reactions were performed in triplicate 20 μL mixture containing 4 μL of 5

×FastPfu Buffer, 2 μL of 2.5 mM dNTPs, 0.8 μL of each primer (5 μM),0.4 μL of FastPfu Polymerase and 10 ng of template DNA and deionized distilled water for a total volume of 20 μl.The amplicon products were loaded on a 2% pre-stained agarose gel (Thermo Fisher Scientific), and the expected fragments were extracted using QuantiFluor™ -ST(Promega, Fitchburg, WI, USA). The final concentration was determined using the Qubit 1X dsDNA HS Assay Kit (Life Technologies, Bleiswijk,Netherlands), and the purified amplicons were normalized and pooled for 2×300 cycles of Illumina MiSeq deep sequencing (Illumina, San Diego,CA, USA).

**Metabolomics profiling**

**Sample preparation**

Serum and ipsilateral brains from the Sham, TBI+saline and TBI+FMT groups (n=9, per group) were collected in sterile and enzyme-free tubes on ice and the brain tissue samples were quickly frozen in liquid nitrogen.The serum sample were thawed in an ice-bath, and 150 µL serum sample was pipetted into a pre-cooled micro high-speed centrifuge tube and mixed with 400 µL pre-chilled methanol/ acetonitrile (v/v = 1/1) and centrifuged at 14,000 g, 4°C for 20 min. The supernatant was dried in a vacuum centrifuge and re-dissolved in 100 μL acetonitrile/water (1:1, v/v) solvent for the UHPLC-Q-TOF/MS analysis.

The brain tissues were cut on dry ice (~10 mg) and transferred into an Eppendorf tube with 200 μL of H_2_O and five ceramic beads for homogenization. Metabolite extraction was performed by adding 800 μL of methanol/acetonitrile (1:1, v/v) to the homogenized solution and centrifuged for 15 min (14000g, 4°C). The supernatant was dried and re-dissolved in 100μL acetonitrile/water (1:1, v/v) solvent for UHPLC-Q-TOF/MS analysis.

**UHPLC-Q-TOF/MS analysis**

the prepared sample solution were analyzed using a 2.1 mm ×100 mm ACQUIY UPLC BEH 1.7µm column. Column temperature was set at 25°C for operation and flow rate was 500 μL/min.The mobile phase consisted of mobile phase A(H_2_O + 25 mM ammonium acetate+25 mM ammonium hydroxide) and mobile phase B (acetonitrile).The gradient was as follows: 95% B from 0 to 0.5 min; 95%−65% B from 0.5 to 7 min; 65%−40% B from 7 to 8 min; B was held at 40% from 8 to 9 min; 40%- 95% B from 9 to 9.1 min; B was held at 95% from 9.1 to 12 min for equilibration. The sample temperature was maintained at 4 °C throughout the analyses. Electrospray ionization source conditions on triple TOF were set as follows: Ion Source Gas1 (Gas1) as 60, Ion Source Gas2 (Gas2) as 60,curtain gas (CUR) as 30, source temperature: 600℃, IonSpray Voltage Floating (ISVF)± 5500 V. In MS only acquisition, the instrument was set to acquire over the m/z range 60-1000 Da, and the accumulation time for TOF MS scan was set at 0.20 s/spectra. In auto MS/MS acquisition,the instrument was set to acquire over the m/z range 25-1000 Da, and the accumulation time for product ion scan was set at 0.05 s/spectra. Information-dependent acquisition(IDA), an artificial intelligence-based product ion scan mode, was used to detect and identify MS/MS spectra.The parameters were set as follows: declustering potential, 60 V (+) and-60 V (-); collision energy, 50 V(±15); exclude isotopes within 4 Da,candidate ions to monitor per cycle: 10. The raw MS data generated by UPLC-Q-TOF/MS were converted to MzXML files using ProteoWizard MSConvert and then imported into XCMS software for peak alignment, retention time adjustment, and extraction of peak intensities. Compound identification of metabolites was performed by comparing the accuracy of m/z value (<25 ppm), and MS/MS spectra with an in-house database established with available authentic standards.

**Proteomics**

**Sample preparation for MS**

Serum samples from the TBI+saline and TBI+FMT groups were taken from -80°C, rewarmed, and centrifuged at 12000 g for 10 min, at 4°C. The supernatant was transferred to a new centrifuge tube and the highly abundant proteins were depleted using the Seppro Rat Spin Columns Kit (Thermo Fisher Scientific). The protein concentrations were determined by the bicinchoninic acid assay and the peptides were fractionated by high pH reverse-phase high-performance liquid chromatography (HPLC) using Agilent 300Extend C18 column (5 μm particles, 4.6 mm ID, 250 mm length; Agilent Technologies) after trypsin

digestion. The operation parameters were as follows: the peptide gradient

was 8–32% acetonitrile, pH was 9, the 60 components were separated for

a duration of 60 min, and the peptides were combined into four components and dried by vacuum centrifugation. 2.9.2 LC-MS/MS analysis The trypsin-digested peptides were dissolved in 0.1% formic acid (solvent A) and separated using the EASY-nLC 1000 ultra-HPLC system. Solvent B included an increase from 4% to 8% solvent (0.1% formic acid in 90% acetonitrile) for 5 min, 8–22% for 5–45 min, 22–30% for 45–55 min, 30–90% for 55–57 min, and 90% for 60 min, at a constant flow rateof 400nL/min. The separated peptides were injected into a nanoelectrospray ionization source for ionization and analysis by Orbitrap

Fusion Lumos Mass Spectrometer. The electrospray voltage was 2.0 kV

and peptide precursor ions and their secondary fragments were detected

and analyzed by high resolution Orbitrap MS. The primary MS scan range was set to 385–1500 m/z, the scan resolution was set to 60,000, and

the secondary MS scan range was set to a fixed starting point of 100 m/z

with a resolution set to 15,000. The data acquisition mode used a data-dependent procedure, namely, the first 20 peptide precursor ions

with the highest signal strength entered into the higher energy collision

dissociation collision cell and fragmented with 30% fragmentation energy,followed by sequential MS analysis. The parameters used for MS were as follows: automatic gain control of 5E4, signal threshold of 15000 ions/s,maximum injection of 30 ms, and dynamic exclusion time for tandem mass scans of 30 s to avoid repeated scans of precursor ions.

**Protein identification and relative quantification**

Raw MS files were analyzed using the Maxquant search engine (v.1.5.2.8) and searched against the Rattus norvegicus UniProt database concatenated with the reverse decoy database. Label-free quantitation was conducted using the intensity-based absolute quantification (iBAQ) method. A maximum of two trypsin/P missing cleavages was allowed.The minimum length of peptide and maximum number of peptide modifications were set to 5 and 7, respectively. In the first search and main search, the mass tolerance for precursor ions was set at 20 and 5 ppm, respectively, and the mass tolerance for fragment ions was set to 0.02 Da. The alkylation of cysteine was specified as fixed modification and oxidation of methionine, acetylation modification and deamination of the N-terminus of proteins were specified as variable modifications. The false discovery rate was set to <1%.

**Result**

Fig. S1. The gut microbiota of each group before surgery. A-B. The sequence reads and OTUs of each group before injury. C-D. α-diversity and β-diversity of the rats before injury. E-G. Bar plot analysis of gut microbiota relative abundance of bacterial phyla, family and genus in the rats before surgery. Different colors represent different phyla, family and genus.

Fig. S1


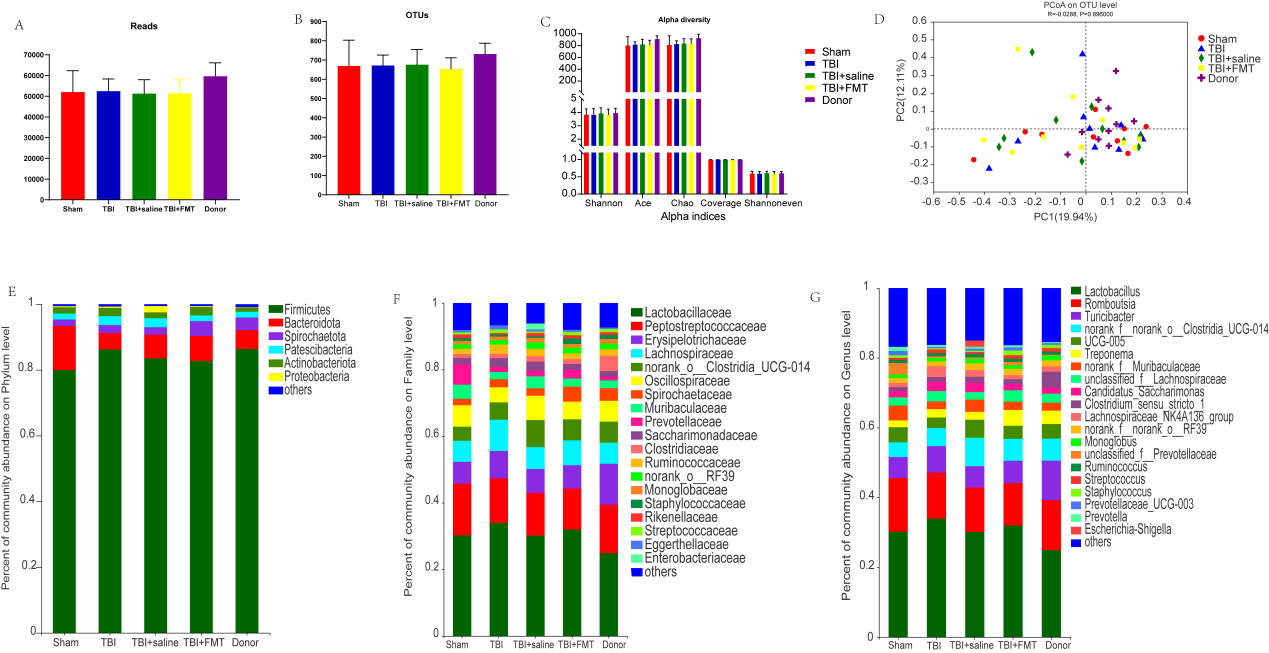


Table S1. The diet composition.

| Component | Value | Component | Value | Component | Value |
| --- | --- | --- | --- | --- | --- |
| Vitamin A | ≥7000IU/Kg | Na | ≥2g/Kg | Protein | ≥180g/Kg |
| Vitamin D | ≥800IU/Kg | K | ≥5g/Kg | Fat | ≥40g/Kg |
| Vitamin E | ≥60IU/Kg | Mg | ≥2g/Kg | Fiber | ≤50g/Kg |
| Vitamin K | ≥3mg/Kg | Cu | ≥10mg/Kg | Lysine | ≥8.2g/Kg |
| Vitamin B1 | ≥8mg/Kg | Fe | ≥100mg/Kg | Methionine+Cystine | ≥5.3g/Kg |
| Vitamin B2 | ≥10mg/Kg | Zn | ≥30mg/Kg | Arginine | ≥9.9g/Kg |
| Vitamin B6 | ≥6mg/Kg | Mn | ≥75mg/Kg | Tryptophan | ≥1.9g/Kg |
| Vitamin B12 | ≥0.02mg/Kg | I | ≥0.5mg/Kg | Histidine | ≥4.0g/Kg |
| Biotin | ≥0.1mg/Kg | Se | ≥0.1-0.2mg/Kg | Phenylpropyl+Tyrosine | ≥11.0g/Kg |
| Nicotinic acid | ≥45mg/Kg | Ca | 10-18g/Kg | Threonine | ≥6.5g/Kg |
| Pantothenic acid | ≥17mg/Kg | P | 6-12g/Kg | Leucine | ≥14.4g/Kg |
| Folic acid | ≥4mg/Kg | Moisture | ≤100g/Kg | Isoleucine | ≥7.0g/Kg |
| Bilineurine | ≥1250mg/Kg | Ash | ≤80g/Kg | Valine | ≥8.4g/Kg |

Table S2. Significant changes of the microbiome at family level among Sham, TBI, TBI+saline and TBI+FMT groups.

| Species name | Sham-Mean±SD(%) | TBI-Mean±SD(%) | TBI+saline-Mean±SD(%) | TBI+FMT-Mean±SD(%) | Corrected pvalue |
| --- | --- | --- | --- | --- | --- |
| f__Lactobacillaceae | 37.45±16.25 | 4.971±5.054 | 3.477±5.815 | 22.85±17.78 | <0.001 |
| f__Enterobacteriaceae | 0.018±0.024 | 30.46±34.33 | 34.46±27.82 | 0.030±0.030 | 0.007 |
| f__Peptostreptococcaceae | 19.09±10.35 | 3.055±4.146 | 1.629±1.992 | 16.72±9.525 | <0.001 |
| f__Erysipelotrichaceae | 6.847±4.704 | 2.457±2.288 | 2.413±2.486 | 12.2±8.081 | 0.012 |
| f__Oscillospiraceae | 5.406±2.508 | 3.541±3.967 | 0.924±1.171 | 6.81±5.403 | 0.002 |
| f__Muribaculaceae | 1.23±0.907 | 7.066±5.825 | 5.054±5.192 | 3.257±1.287 | 0.005 |
| f__Bacteroidaceae | 0.178±0.184 | 9.839±15.11 | 4.937±6.095 | 0.384±0.2767 | 0.042 |
| f__norank_o__Clostridia_UCG-014 | 6.171±5.037 | 0.040±0.032 | 0.044±0.062 | 5.599±2.757 | 0.000 |
| f__Coriobacteriaceae | 0.284±0.450 | 1.882±1.517 | 8.563±7.648 | 0.164±0.199 | 0.008 |
| f__Erysipelatoclostridiaceae | 0.084±0.064 | 3.577±1.897 | 7.106±6.51 | 0.092±0.097 | 0.001 |
| f__Saccharimonadaceae | 3.793±3.022 | 0.326±0.289 | 0.221±0.206 | 2.2±1.387 | 0.002 |
| f__Clostridiaceae | 0.660±0.755 | 0.851±1.249 | 0.217±0.290 | 3.527±3.068 | 0.033 |
| f__Ruminococcaceae | 1.737±1.081 | 0.298±0.257 | 0.379±0.384 | 2.829±2.725 | 0.007 |
| f__norank_o__RF39 | 2.159±1.527 | 0.031±0.044 | 0.014±0.013 | 1.531±1.442 | 0.003 |
| f__Aerococcaceae | 0.797±0.699 | 0.138±0.186 | 2.102±2.593 | 0.006±0.007 | 0.010 |
| f__Monoglobaceae | 0.512±0.270 | 0.024±0.019 | 0.050±0.0389 | 1.887±1.182 | <0.001 |
| f__Tannerellaceae | 0.073±0.052 | 1.103±1.179 | 1.035±1.456 | 0.131±0.088 | 0.037 |
| f__Streptococcaceae | 1.07±1.123 | 0.010±0.009 | 0.013±0.013 | 0.916±1.047 | 0.027 |
| f__unclassified_p__Firmicutes | 0.745±0.253 | 0.039±0.084 | 0.005±0.014 | 0.707±0.315 | 0.000 |
| f__Corynebacteriaceae | 0.399±0.267 | 0.20±0.202 | 0.608±0.592 | 0.015±0.008 | 0.002 |
| f__p-2534-18B5_gut_group | 0.196±0.331 | 0.0002±0.0001 | 0.091±0.285 | 0.799±0.606 | 0.014 |
| f__Selenomonadaceae | 0.0821±0.053 | 0.021±0.022 | 0.268±0.598 | 0.6743±0.4243 | 0.002 |
| f__Staphylococcaceae | 0.515±0.548 | 0.221±0.223 | 0.2568±0.1808 | 0.013±0.007 | 0.003 |
| f__Christensenellaceae | 0.285±0.114 | 0.079±0.078 | 0.041±0.059 | 0.554±0.285 | <0.001 |
| f__Atopobiaceae | 0.016±0.023 | 0.234±0.185 | 0.555±0.738 | 0.025±0.023 | 0.016 |
| f__unclassified_c__Bacilli | 0.285±0.154 | 0.035±0.065 | 0.004±0.005 | 0.202±0.139 | <0.001 |
| f__unclassified_o__Lactobacillales | 0.251±0.267 | 0.001±0.002 | 0.042±0.057 | 0.1214±0.1545 | 0.020 |
| f__Sutterellaceae | 0.008±0.0071 | 0.236±0.263 | 0.102±0.151 | 0.061±0.062 | 0.024 |
| f__norank_o__Gastranaerophilales | 0.080±0.053 | 0.009±0.007 | 0.020±0.015 | 0.222±0.182 | 0.002 |
| f__unclassified_c__Clostridia | 0.123±0.173 | 0.009±0.014 | 0.005±0.005 | 0.158±0.1121 | 0.009 |
| f__unclassified_o__Coriobacteriales | 0.002±0.001 | 0.0858±0.104 | 0.113±0.085 | 0.011±0.001 | 0.003 |
| f__unclassified_o__Oscillospirales | 0.020±0.01 | 0.008±0.011 | 0.013±0.020 | 0.040±0.028 | 0.037 |
| f__Burkholderiaceae | 0.004±0.006 | 0.011±0.012 | 0.014±0.011 | 0.003±0.003 | 0.039 |
| f__Micrococcaceae | 0.018±0.013 | 0.004±0.009 | 0.003±0.002 | 0.008±0.001 | 0.035 |
| f__Peptococcaceae | 0.01±0.014 | 0.003±0.005 | 0.0004±0.001 | 0.014±0.014 | 0.033 |
| f__Xanthobacteraceae | 0.001±0.002 | 0.004±0.006 | 0.006±0.004 | 0.008±0.006 | 0.033 |
| f__Dietziaceae | 0.004±0.004 | 0.004±0.004 | 0.006±0.006 | 0.0005±0.001 | 0.006 |
| f__Dermabacteraceae | 0.005±0.005 | 0.0029±0.005 | 0.003±0.005 | 0.0002±0.0007 | 0.040 |
| f__unclassified_c__Coriobacteriia | 0.003±0.004 | 0.001±0.002 | 0.0002±0.001 | 0.004±0.004 | 0.043 |

Table S3. Significant changes of the microbiome at genus level among Sham, TBI, TBI+saline and TBI+FMT groups.

| Species name | Sham-Mean±SD(%) | TBI-Mean±SD(%) | TBI+saline-Mean±SD(%) | TBI+FMT-Mean±SD(%) | Corrected pvalue |
| --- | --- | --- | --- | --- | --- |
| g__Lactobacillus | 37.45±16.25 | 4.971±5.054 | 3.477±5.815 | 22.85±17.78 | 0.001 |
| g__Romboutsia | 18.94±10.37 | 3.034±4.129 | 1.591±1.985 | 16.51±9.47 | 0.001 |
| g__Escherichia-Shigella | 0.017±0.024 | 12.82±11.15 | 23.75±23.84 | 0.03±0.029 | 0.011 |
| g__norank_f__Muribaculaceae | 1.23±0.907 | 7.066±5.825 | 5.054±5.192 | 3.257±1.287 | 0.007 |
| g__Turicibacter | 5.86±4.802 | 1.469±2.184 | 0.504±0.657 | 7.88±5.61 | 0.007 |
| g__Bacteroides | 0.178±0.184 | 9.839±15.11 | 4.937±6.095 | 0.384±0.277 | 0.050 |
| g__norank_f__norank_o__Clostridia_UCG-014 | 6.171±5.037 | 0.040±0.032 | 0.044±0.062 | 5.599±2.757 | 0.001 |
| g__Collinsella | 0.284±0.450 | 1.882±1.517 | 8.563±7.648 | 0.164±0.199 | 0.011 |
| g__UCG-005 | 4.422±2.364 | 0.023±0.023 | 0.062±0.184 | 4.146±4.791 | 0.003 |
| g__Candidatus_Stoquefichus | 0.016±0.022 | 1.647±1.5 | 6.041±6.324 | 0.002±0.003 | 0.012 |
| g__Candidatus_Saccharimonas | 3.793±3.022 | 0.326±0.289 | 0.221±0.206 | 2.2±1.387 | 0.004 |
| g__Clostridium_sensu_stricto_1 | 0.660±0.755 | 0.851±1.249 | 0.217±0.291 | 3.526±3.069 | 0.037 |
| g__Adlercreutzia | 0.136±0.115 | 1.628±1.673 | 1.819±1.927 | 0.249±0.172 | 0.027 |
| g__norank_f__norank_o__RF39 | 2.159±1.527 | 0.031±0.044 | 0.014±0.013 | 1.531±1.442 | 0.006 |
| g__Aerococcus | 0.735±0.626 | 0.134±0.184 | 2.097±2.591 | 0.005±0.006 | 0.012 |
| g__Monoglobus | 0.512±0.270 | 0.024±0.019 | 0.050±0.039 | 1.887±1.182 | 0.001 |
| g__Parabacteroides | 0.073±0.053 | 1.103±1.179 | 1.035±1.456 | 0.1310.088 | 0.045 |
| g__Streptococcus | 1.07±1.123 | 0.01±0.009 | 0.013±0.013 | 0.916±1.047 | 0.032 |
| g__norank_f__Ruminococcaceae | 0.548±0.345 | 0.121±0.114 | 0.106±0.222 | 1.178±2.106 | 0.032 |
| g__Lachnospiraceae_NK4A136_group | 0.854±0.947 | 0.004±0.004 | 0.006±0.009 | 0.992±0.727 | 0.009 |
| g__Ruminococcus | 0.708±0.449 | 0.009±0.011 | 0.006±0.006 | 1.126±1.106 | 0.004 |
| g__unclassified_f__Oscillospiraceae | 0.265±0.246 | 0.499±0.731 | 0.075±0.129 | 0.885±0.574 | 0.011 |
| g__unclassified_p__Firmicutes | 0.745±0.253 | 0.039±0.084 | 0.005±0.014 | 0.707±0.315 | <0.001 |
| g__Corynebacterium | 0.399±0.266 | 0.20±0.202 | 0.608±0.592 | 0.015±0.008 | 0.004 |
| g__norank_f__p-2534-18B5_gut_group | 0.196±0.331 | 0.0002±0.001 | 0.091±0.285 | 0.799±0.606 | 0.017 |
| g__Quinella | 0.082±0.054 | 0.018±0.019 | 0.261±0.596 | 0.673±0.425 | 0.004 |
| g__NK4A214_group | 0.208±0.172 | 0.106±0.248 | 0.024±0.038 | 0.555±0.504 | 0.015 |
| g__Coriobacteriaceae_UCG-002 | 0.010±0.008 | 0.234±0.185 | 0.555±0.737 | 0.025±0.023 | 0.014 |
| g__Christensenellaceae_R-7_group | 0.259±0.104 | 0.009±0.009 | 0.012±0.029 | 0.481±0.281 | 0.000 |
| g__Eubacterium_xylanophilum_group | 0.243±0.332 | 0.0002±0.0006 | 0.002±0.002 | 0.478±0.826 | 0.045 |
| g__Staphylococcus | 0.348±0.513 | 0.164±0.175 | 0.173±0.108 | 0.008±0.005 | 0.005 |
| g__Marvinbryantia | 0.454±0.363 | 0.011±0.014 | 0.007±0.012 | 0.175±0.091 | 0.001 |
| g__Mogibacterium | 0.013±0.014 | 0.25±0.263 | 0.322±0.347 | 0.003±0.005 | 0.016 |
| g__unclassified_c__Bacilli | 0.285±0.154 | 0.035±0.065 | 0.0041±0.005 | 0.207±0.139 | 0.001 |
| g__Ruminococcus_gauvreauii_group | 0.119±0.190 | 0.124±0.281 | 0.015±0.042 | 0.264±0.152 | 0.009 |
| g__unclassified_o__Lactobacillales | 0.251±0.267 | 0.001±0.002 | 0.042±0.057 | 0.121±0.154 | 0.025 |
| g__Parasutterella | 0.008±0.007 | 0.233±0.264 | 0.091±0.135 | 0.064±0.06 | 0.031 |
| g__unclassified_f__Peptostreptococcaceae | 0.146±0.054 | 0.018±0.029 | 0.004±0.004 | 0.211±0.082 | 0.000 |
| g__norank_f__Lachnospiraceae | 0.165±0.225 | 0.017±0.032 | 0.009±0.016 | 0.18±0.131 | 0.015 |
| g__Roseburia | 0.146±0.136 | 0.004±0.004 | 0.004±0.001 | 0.209±0.212 | 0.017 |
| g__norank_f__norank_o__Gastranaerophilales | 0.08±0.053 | 0.009±0.007 | 0.021±0.015 | 0.222±0.182 | 0.004 |
| g__unclassified_c__Clostridia | 0.123±0.173 | 0.009±0.014 | 0.005±0.005 | 0.158±0.112 | 0.012 |
| g__Jeotgalicoccus | 0.146±0.074 | 0.056±0.106 | 0.083±0.113 | 0.005±0.004 | 0.003 |
| g__Odoribacter | 0.043±0.036 | 0.027±0.03 | 0.002±0.005 | 0.148±0.152 | 0.009 |
| g__unclassified_o__Coriobacteriales | 0.002±0.0012 | 0.086±0.104 | 0.113±0.085 | 0.011±0.009 | 0.005 |
| g__unclassified_f__Ruminococcaceae | 0.067±0.058 | 0.0269±0.03 | 0.014±0.02 | 0.089±0.054 | 0.012 |
| g__Candidatus_Soleaferrea | 0.005±0.004 | 0.042±0.043 | 0.138±0.168 | 0.004±0.006 | 0.048 |
| g__Parvibacter | 0.006±0.004 | 0.079±0.082 | 0.025±0.020 | 0.025±0.021 | 0.015 |
| g__Rikenella | 0.045±0.042 | 0.003±0.005 | 0.001±0.001 | 0.0814±0.1 | 0.025 |
| g__Frisingicoccus | 0.001±0.002 | 0.078±0.098 | 0.021±0.029 | 0.013±0.013 | 0.030 |
| g__Family_XIII_AD3011_group | 0.030±0.020 | 0.061±0.101 | 0.004±0.014 | 0.019±0.014 | 0.037 |
| g__unclassified_f__Anaerovoracaceae | 0.030±0.016 | 0.032±0.074 | 0.007±0.006 | 0.031±0.022 | 0.007 |
| g__unclassified_o__Oscillospirales | 0.020±0.01 | 0.008±0.011 | 0.013±0.02 | 0.040±0.028 | 0.045 |
| g__Facklamia | 0.060±0.10 | 0.003±0.004 | 0.006±0.006 | 0.0002±0.0007 | 0.046 |
| g__UCG-009 | 0.002±0.004 | 0.045±0.0444 | 0.005±0.007 | 0.0148±0.016 | 0.039 |
| g__Eubacterium_brachy_group | 0.013±0.010 | 0.018±0.04 | 0.002±0.002 | 0.026±0.024 | 0.015 |
| g__Tuzzerella | 0.013±0.009 | 0.005±0.004 | 0.006±0.006 | 0.022±0.013 | 0.016 |
| g__Dielma | 0.001±0.002 | 0.024±0.0237 | 0.007±0.012 | 0.005±0.004 | 0.037 |
| g__UCG-007 | 0.006±0.005 | 0.0003±0.0007 | 0.002±0.006 | 0.0253±0.022 | 0.009 |
| g__norank_f__Peptococcaceae | 0.009±0.014 | 0.003±0.005 | 0.0002±0.001 | 0.014±0.014 | 0.036 |
| g__unclassified_f__Christensenellaceae | 0.0008±0.002 | 0.005±0.006 | 0.002±0.003 | 0.009±0.007 | 0.046 |
| g__Dietzia | 0.004±0.004 | 0.004±0.004 | 0.006±0.006 | 0.0005±0.001 | 0.009 |
| g__Bradyrhizobium | 0.001±0.002 | 0.004±0.006 | 0.006±0.004 | 0.004±0.003 | 0.032 |
| g__Brachybacterium | 0.005±0.005 | 0.003±0.005 | 0.003±0.006 | 0.0002±0.0007 | 0.048 |
| g__Harryflintia | 0.001±0.002 | 0.002±0.003 | 0.0004±0.001 | 0.004±0.003 | 0.048 |

Table S4. The significantly differential brain metabolites between TBI+saline and Sham groups.

| Metabolites | rt(s) | m/z | VIP | FC | p-value |
| --- | --- | --- | --- | --- | --- |
| L-Carnitine | 361.106 | 162.112 | 44.016 | 2.859 | <0.001 |
| 2-Keto-D-gluconic acid | 233.976 | 159.027 | 3.483 | 0.726 | <0.001 |
| 1-Stearoyl-2-hydroxy-sn-glycero-3-phosphocholine | 187.826 | 568.339 | 6.770 | 0.511 | <0.001 |
| Larixinic Acid | 478.552 | 127.037 | 3.255 | 3.331 | <0.001 |
| 4-Hydroxybutanoic acid lactone | 376.945 | 87.044 | 6.441 | 0.759 | <0.001 |
| Acetylcarnitine | 333.570 | 204.122 | 2.209 | 6.347 | <0.001 |
| D-Ribulose 5-phosphate | 449.107 | 213.014 | 1.943 | 2.618 | <0.001 |
| Glutathione disulfide | 499.799 | 613.157 | 1.991 | 2.109 | <0.001 |
| Vanillin | 469.473 | 219.004 | 1.757 | 3.378 | <0.001 |
| 4-Aminobutyric acid | 376.883 | 104.070 | 4.029 | 0.830 | <0.001 |
| Creatinine | 173.850 | 114.065 | 3.615 | 1.256 | <0.001 |
| Deoxycytidine | 210.869 | 228.097 | 4.181 | 2.139 | <0.001 |
| L-Serine | 399.827 | 106.048 | 1.710 | 2.218 | <0.001 |
| D-Glucose 6-phosphate | 478.474 | 278.062 | 2.040 | 2.508 | <0.001 |
| Xanthine | 225.039 | 153.039 | 1.270 | 1.392 | <0.001 |
| Stearoylcarnitine | 149.761 | 428.371 | 3.584 | 1.985 | <0.001 |
| 5-Methylcytosine | 201.515 | 126.065 | 1.011 | 3.140 | <0.001 |
| 2-Methylbutyroylcarnitine | 241.795 | 246.169 | 12.788 | 39.366 | <0.001 |
| L-Palmitoylcarnitine | 172.744 | 400.341 | 8.693 | 2.961 | <0.001 |
| N-Acetyl-L-aspartic acid | 400.372 | 176.055 | 3.370 | 0.652 | <0.001 |
| D-Mannose-6-phosphate | 477.920 | 261.036 | 5.310 | 2.733 | <0.001 |
| alpha-D-Glucose 1-phosphate | 478.397 | 243.025 | 5.497 | 3.558 | <0.001 |
| Pro-Glu | 419.587 | 245.112 | 1.533 | 1.340 | <0.001 |
| Allopurinol riboside | 221.940 | 269.087 | 3.788 | 1.595 | <0.001 |
| Creatine | 390.042 | 132.076 | 6.895 | 0.768 | 0.001 |
| (3-Carboxypropyl)trimethylammonium cation | 380.851 | 146.117 | 8.428 | 1.412 | 0.001 |
| L-Methionine | 299.131 | 150.057 | 1.232 | 1.565 | 0.001 |
| Thioetheramide-PC | 90.949 | 758.569 | 9.220 | 1.702 | 0.001 |
| Trimethylamine N-oxide | 333.965 | 76.075 | 1.928 | 14.146 | 0.001 |
| L-Pyroglutamic acid | 424.879 | 147.076 | 3.476 | 1.266 | 0.002 |
| 4-Guanidinobutyric acid | 367.732 | 146.091 | 2.031 | 0.660 | 0.002 |
| Anthranilic acid (Vitamin L1) | 334.926 | 138.053 | 1.037 | 4.232 | 0.002 |
| Tyramine | 277.753 | 120.079 | 4.366 | 1.741 | 0.002 |
| L-Leucine | 282.936 | 132.100 | 3.861 | 1.593 | 0.002 |
| N-Oleoylethanolamine | 36.069 | 326.304 | 3.157 | 0.691 | 0.003 |
| D-Proline | 323.113 | 116.070 | 1.729 | 1.325 | 0.004 |
| Cyclohexylamine | 388.461 | 160.132 | 9.374 | 6.809 | 0.004 |
| Hypoxanthine | 175.157 | 137.046 | 20.605 | 0.907 | 0.005 |
| O-Acetyl-L-serine | 94.826 | 148.059 | 1.205 | 0.696 | 0.005 |
| Prostaglandin D2(PGD2) | 119.722 | 370.256 | 1.028 | 1.516 | 0.006 |
| 1-Palmitoyl-sn-glycero-3-phosphocholine | 193.076 | 496.340 | 3.567 | 1.074 | 0.021 |
| PC(16:0/16:0) | 147.838 | 734.569 | 7.800 | 0.752 | 0.022 |
| Nitrobenzene | 64.793 | 106.027 | 1.031 | 0.908 | 0.022 |
| Thiamine | 370.909 | 265.110 | 1.232 | 0.674 | 0.032 |
| Pantothenate | 280.393 | 220.116 | 1.021 | 1.171 | 0.036 |
| Betaine | 391.139 | 118.085 | 1.832 | 0.753 | 0.047 |
| Diacetyl | 193.578 | 87.043 | 1.371 | 0.718 | 0.062 |
| Acetylcholine | 193.462 | 146.116 | 3.172 | 0.708 | 0.063 |
| 1,2-dioleoyl-sn-glycero-3-phosphatidylcholine | 89.737 | 808.581 | 2.183 | 1.130 | 0.073 |
| 3-Methylhistidine | 415.146 | 170.091 | 1.309 | 1.368 | 0.075 |
| 1-O-(cis-9-Octadecenyl)-2-O-acetyl-sn-glycero-3-phosphocholine | 187.826 | 550.384 | 1.196 | 0.845 | 0.090 |

Table S5. The significantly differential serum metabolites between TBI+saline and Sham groups.

| Metabolites | rt(s) | m/z | VIP | FC | p-value |
| --- | --- | --- | --- | --- | --- |
| Sphingosine | 48.241 | 300.288 | 1.582 | 3.069 | 0.001 |
| L-Isoleucine | 247.976 | 173.127 | 2.462 | 0.658 | 0.001 |
| N-Palmitoylsphingosine | 34.593 | 538.518 | 3.127 | 2.280 | 0.002 |
| Glycochenodeoxycholate | 215.888 | 450.318 | 1.410 | 3.327 | 0.003 |
| L-Citrulline | 444.719 | 176.101 | 1.082 | 0.680 | 0.005 |
| L-Pipecolic acid | 271.096 | 171.112 | 3.958 | 0.731 | 0.005 |
| 1-Myristoyl-sn-glycero-3-phosphocholine | 199.096 | 468.307 | 2.543 | 0.683 | 0.013 |
| L-Phenylalanine | 282.802 | 166.084 | 1.181 | 1.174 | 0.022 |
| 20-Hydroxyarachidonic acid | 36.143 | 303.230 | 1.148 | 2.209 | 0.030 |
| Trimethylamine N-oxide | 333.593 | 76.074 | 1.953 | 3.936 | 0.035 |
| 1-Palmitoyllysophosphatidylcholine | 189.717 | 538.384 | 1.891 | 0.816 | 0.035 |
| 1-O-(cis-9-Octadecenyl)-2-O-acetyl-sn-glycero-3-phosphocholine | 189.098 | 550.384 | 2.461 | 0.822 | 0.037 |
| 1-Palmitoyl-2-hydroxy-sn-glycero-3-phosphoethanolamine | 202.338 | 454.292 | 2.525 | 1.550 | 0.038 |
| 1-Palmitoyl-sn-glycero-3-phosphocholine | 194.741 | 496.340 | 18.411 | 0.896 | 0.046 |
| L-Anserine | 424.599 | 241.128 | 2.132 | 0.326 | 0.056 |
| 1-Aminocyclopropanecarboxylic acid | 443.912 | 84.044 | 1.484 | 0.754 | 0.057 |

Abbreviations: FC, fold change; m/z, mass-to-charge ratio; Rt, retention time; VIP, variable importance in the projection.

Table S6. The significantly differential serum protein between TBI+saline and TBI+FMT groups.

| Accession | Description | Gene  name | Coverage (%) | MW (kDa) | FMT/TBI Ratio | Regulation |
| --- | --- | --- | --- | --- | --- | --- |
| A0A0G2K477 | Immunoglobulin heavy constant mu | Ighm | 24.9 | 51.136 | 2.614 | Up |
| Q6AXN2 | EGF-containing fibulin-like extracellular matrix protein 1 | Efemp1 | 24.3 | 54.654 | 0.377 | Down |
| Q5M872 | Dipeptidase 2 | Dpep2 | 15.2 | 53.284 | 0.147 | Down |
| O35244 | Peroxiredoxin-6 | Prdx6 | 16.1 | 24.818 | 2.116 | Up |
| B2RZ72 | Actin-related protein 2/3 complex subunit 4 | Arpc4 | 11.3 | 19.667 | 0.622 | Down |
| Q06000 | Lipoprotein lipase | Lpl | 9.1 | 53.082 | 0.377 | Down |
| P05197 | Elongation factor 2 | Eef2 | 4.2 | 95.283 | 0.464 | Down |
| F1M7X5 | Dipeptidyl peptidase 4 | Dpp4 | 8 | 88.053 | 2.817 | Up |
| O70513 | Galectin-3-binding protein | Lgals3bp | 3.7 | 63.742 | 0.487 | Down |
| A0A0G2JW10 | Receptor protein-tyrosine kinase | Kdr | 7.1 | 110.65 | 3.143 | Up |
| P20762 | Ig gamma-2C chain C region | --- | 26.7 | 36.571 | 2.577 | Up |
| P27139 | Carbonic anhydrase 2 | Ca2 | 44.6 | 29.113 | 3.566 | Up |
| F1MAE7 | Uncharacterized protein | --- | 12.8 | 12.26 | 2.089 | Up |
| F7EY63 | L-selectin | Sell | 16.3 | 49.103 | 0.634 | Down |
| Q4FZZ3 | Glutathione S-transferase alpha-5 | Gsta5 | 8.1 | 25.569 | 3.542 | Up |
| F1M6W1 | Fumarylacetoacetase | Fah | 9 | 46.081 | 0.206 | Down |
| P05065 | Fructose-bisphosphate aldolase A | Aldoa | 64.8 | 39.351 | 3.251 | Up |
| D3ZUL3 | Collagen type VI alpha 1 chain | Col6a1 | 4.7 | 108.8 | 2.318 | Up |
| A0A0G2JSH9 | Peroxiredoxin-2 | Prdx2 | 31.8 | 21.797 | 2.727 | Up |
| P02600 | Myosin light chain 1/3, skeletal muscle isoform | Myl1 | 41.3 | 20.679 | 4.241 | Up |
| Q99J86 | Attractin | Atrn | 18.1 | 158.67 | 1.703 | Up |
| A0A0G2JSZ3 | Neuroblastoma suppressor of tumorigenicity 1 | Nbl1 | 6.2 | 19.144 | 4.224 | Up |
